# Enhancing antitumor efficacy of CLDN18.2-directed antibody-drug conjugates through autophagy inhibition in gastric cancer

**DOI:** 10.1038/s41420-024-02167-0

**Published:** 2024-09-03

**Authors:** Wenjing Xue, Caili Xu, Kaiqi Zhang, Lu Cui, Xiting Huang, Yanyang Nan, Dianwen Ju, Xusheng Chang, Xuyao Zhang

**Affiliations:** 1 Department of Biological Medicines & Shanghai Engineering Research Center of Immunotherapeutics, School of Pharmacy, Fudan University, Shanghai, 201203 China; 2 Department of Gastrointestinal Surgery, Changhai Hospital, Naval Medical University, Shanghai, 200433 China

**Keywords:** Gastric cancer, Cancer therapy

## Abstract

Claudin18.2 (CLDN18.2) is overexpressed in cancers of the digestive system, rendering it an ideal drug target for antibody-drug conjugates (ADCs). Despite many CLDN18.2-directed ADCs undergoing clinical trials, the inconclusive underlying mechanisms pose a hurdle to extending the utility of these agents. In our study, αCLDN18.2-MMAE, an ADC composed of an anti-CLDN18.2 monoclonal antibody and the tubulin inhibitor MMAE, induced a dose-dependent apoptosis via the cleavage of caspase-9/PARP proteins in CLDN18.2-positive gastric cancer cells. It was worth noting that autophagy was remarkably activated during the αCLDN18.2-MMAE treatment, which was characterized by the accumulation of autophagosomes, the conversion of autophagy marker LC3 from its form I to II, and the complete autophagic flux. Inhibiting autophagy by autophagy inhibitor LY294002 remarkably enhanced αCLDN18.2-MMAE-induced cytotoxicity and caspase-mediated apoptosis, indicating the cytoprotective role of autophagy in CLDN18.2-directed ADC-treated gastric cancer cells. Combination with an autophagy inhibitor significantly potentiated the in vivo antitumoral efficacy of αCLDN18.2-MMAE. Besides, the Akt/mTOR pathway inactivation was demonstrated to be implicated in the autophagy initiation in αCLDN18.2-MMAE-treated gastric cancer cells. In conclusion, our study highlighted a groundbreaking investigation into the mechanism of the CLDN18.2-directed ADC, focusing on the crucial role of autophagy, providing a novel insight to treat gastric cancer by the combination of CLDN18.2-directed ADC and autophagy inhibitor.

## Introduction

Gastric cancer is one of the most common cancers of the digestive system [1]. Due to insidious symptoms and inadequate screening systems, patients are often diagnosed in advanced stages when surgical resection becomes unfeasible. Chemotherapy, primarily based on platinum and fluoropyrimidine, stands as the mainstay treatment for advanced and metastatic gastric cancer [2, 3]. However, it encounters challenges such as drug resistance and adverse effects, leading to a median overall survival of about 12 months [4]. Hence, there is an urgent imperative to explore novel therapeutics.

Antibody-drug conjugates (ADCs) involve the conjugation of an antibody and cytotoxic agents through cleavable linkers, proving to be a promising targeted therapy [5]. The promising targets of ADCs to treat gastric cancer mainly include HER2, Claudin18.2 (CLDN18.2), and EGFR [6–9]. Among them, CLDN18.2 has garnered much attention due to its specific and high expression in gastric cancer. CLDN18.2, a member of the Claudin protein family, typically features four transmembrane domains and two extracellular loops [10, 11]. In normal tissues, CLDN18.2 epitopes, though present, are typically concealed by tight junctions, impeding their exposure. However, the disruption of tight junctions by malignant tumors facilitates the binding of antibody drugs [12]. Zolbetuximab, an IgG1-type antibody targeting CLDN18.2, has demonstrated efficacy and safety in an international phase III clinical trial for CLDN18.2-positive, HER2-negative, unresectable or metastatic gastric cancer [13]. In this trial, the combined use of zolbetuximab and chemotherapy significantly reduced the risk of disease progression and mortality. The median progression-free survival for the zolbetuximab group was 10.61 months, compared to 8.67 months for the placebo group. The efficacy and safety of zolbetuximab underscored the potential of CLDN18.2 as an encouraging therapeutic target for gastric cancer. According to information from both clinicaltrials.gov, and the drug clinical trial registration and information publicity platform, a dozen ADCs targeting CLDN18.2 are currently undergoing clinical trials, including SKB315, RC118, ATG022, LM302, SYSA1801, IBI343, AZD0901, TQB2103, EO-3021, and JS107. Despite the progress in clinical trials, the underlying mechanisms of these agents are not yet fully understood, posing a significant challenge to expanding the scope of their application.

Autophagy is a critical intracellular degradation process in eukaryotic cells, responsible for the clearance of senescent proteins and malfunctioning organelles, thus maintaining cellular homeostasis [14, 15]. In cancer treatment, the role of autophagy is multifaceted and contingent upon tumor types and stages of tumorigenesis [16, 17]. On one hand, autophagy serves as a tumor-suppressive mechanism by eliminating integrant cellular components. On the other hand, it exhibits a cytoprotective role, facilitating cancer cell survival and adaptation to harsh microenvironments characterized by hypoxia and nutrient scarcity. Several studies have shown that certain drugs such as asparaginase, signal regulatory protein α-Fc and cisplatin used in cancer treatment can induce autophagy [18–20]. The combination therapy of autophagy inhibitors with certain antitumor drugs can significantly enhance their efficacy [21–23]. In non-small cell lung cancer models, autophagy inhibitor chloroquine significantly enhanced the effectiveness of erlotinib in inhibiting tumor growth and suppressing the JAK2/STAT3/VEGFA pathway [24]. However, it remains unclear whether and how autophagy influences CLDN18.2-targeted ADC therapy in gastric cancer, highlighting the need for further investigation.

In this study, we delved into the potential impacts of αCLDN18.2-MMAE on CLDN18.2-positive gastric cancer cells, and it was the first report that we found autophagy was triggered during αCLDN18.2-MMAE-induced caspase-dependent apoptosis in gastric cancer cells. We further elucidated the cytoprotective role and underlying mechanism of autophagy induced by αCLDN18.2-directed ADCs. In addition, inhibiting autophagy by LY294002 significantly potentiates the in vivo antitumor effects of αCLDN18.2-MMAE. Our findings underscored the potential of combining CLDN18.2-directed ADCs with autophagy inhibitors as a novel and encouraging therapeutic approach to treat gastric cancer.

## Results

### αCLDN18.2-MMAE demonstrated significant antitumor efficacy against CLDN18.2-positive gastric cancer cells

**Fig. 1:**
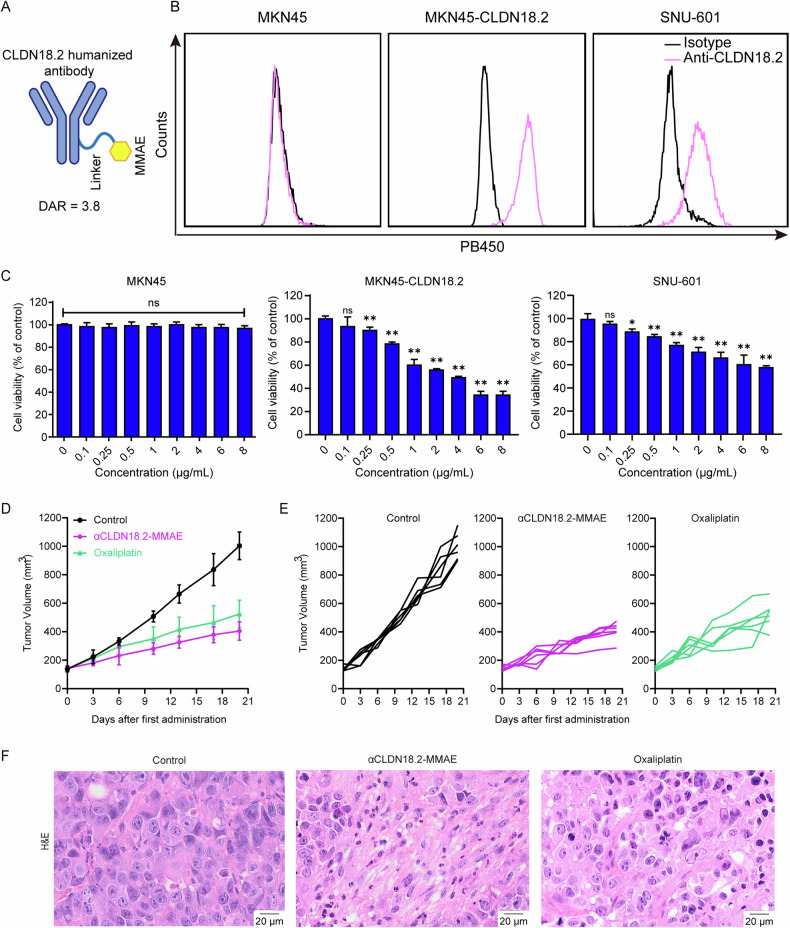
αCLDN18.2-MMAE demonstrated significant antitumor efficacy against CLDN18.2-positive gastric cancer cells. **A** Structure of αCLDN18.2-MMAE **B** The expression level of CLDN18.2 on three human gastric cancer cell lines was determined by flow cytometry. **C** To evaluate the cytotoxicity of αCLDN18.2-MMAE on CLDN18.2-positive gastric cancer cells, the cell viability was assessed using the CCK-8 assay after treatment with cumulative concentrations of αCLDN18.2-MMAE for 48 h. The results were presented as mean ± S.D. (*n* = 3) and analyzed by two-tailed unpaired t-test (ns not significant, *P < 0.05, **P < 0.01). **D**, **E** Human gastric cancer xenograft models were established by subcutaneous injection of MKN45-CLDN18.2 cells into NCG mice. Mice were administered 2 mg/kg of αCLDN18.2-MMAE or 5 mg/kg of oxaliplatin twice weekly. The tumor volume was measured and calculated using length × width^2^/2. The tumor volume data were shown as mean ± S.D. (*n* = 6). **F** H&E staining of tumor tissues from the indicated treatment. Scale bar = 20 μm.

To assess the selective cytotoxicity of αCLDN18.2-MMAE in vitro, we screened out CLDN18.2-positive gastric cancer cell lines (SNU-601 and MKN45-CLDN18.2), the CLDN18.2-negative MKN45 (Fig. 1A, B) and CLDN18.2-negative human normal gastric mucosal epithelial cell line GES-1 (Fig. S1A).αCLDN18.2-MMAE showed dose-dependent cytotoxicity against MKN45-CLDN18.2 and SNU-601 cells, reducing the cell viability to approximately 50 and 66% respectively under a drug concentration of 4 μg/mL for 48 h (Fig. 1C). Contrastively, minimal cytotoxicity was observed against MKN45 and GES-1, indicating its specificity for target cells (Figs. 1C and S1B). Next, we evaluated the in vivo antitumor efficacy of αCLDN18.2-MMAE. MKN45-CLDN18.2 cells were implanted into NCG mice to establish a subcutaneous xenograft model. Subsequently, mice were randomly assigned to the negative control, αCLDN18.2-MMAE (2 mg/kg), and oxaliplatin (5 mg/kg). Tumor volumes were regularly measured. Compared to the negative control group, mice treated with αCLDN18.2-MMAE exhibited a significant reduction in tumor volume, surpassing the therapeutic efficacy observed in the positive control group treated with oxaliplatin (Fig. 1D, E). H&E staining revealed increased necrosis in tumor tissues treated with αCLDN18.2-MMAE (Fig. 1F). These findings indicated that αCLDN18.2-MMAE induced potent cytotoxicity against CLDN18.2-positive gastric cancer cells both in vitro and in vivo.

### Apoptosis was activated by αCLDN18.2-MMAE in CLDN18.2-positive gastric cancer cells

**Fig. 2:**
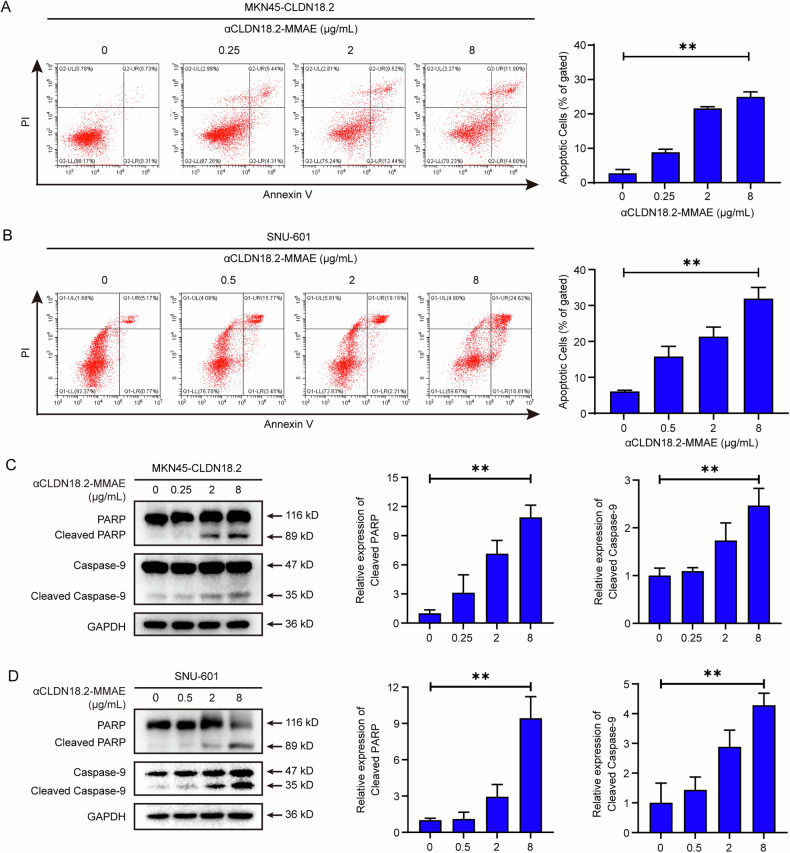
Apoptosis was activated by αCLDN18.2-MMAE in CLDN18.2-positive gastric cancer cells. **A**, **B** MKN45-CLDN18.2 and SNU-601 cells were incubated with indicated concentrations of αCLDN18.2-MMAE for 48 h and then stained with Annexin V-FITC/PI to detect apoptosis by flow cytometry. The proportions of Annexin V-positive cells from repeat experiments were statistically calculated. Data were shown as mean ± S.D. (*n* = 3) and analyzed by ordinary one-way ANOVA (**P < 0.01). **C**, **D** After 48 h of treatment with varying concentrations of αCLDN18.2-MMAE, the expression levels of cleaved PARP and cleaved caspase-9 were determined through Western Blot analysis and quantified by ImageJ. The results were presented as mean ± S.D. (*n* = 3) and analyzed by ordinary one-way ANOVA (**P < 0.01).

Considering apoptosis as a pivotal form of cell death, we extended our investigation into the ability of αCLDN18.2-MMAE to induce apoptosis specifically in CLDN18.2-positive gastric cancer cells. After co-incubating MKN45-CLDN18.2 and SNU-601 cells with varying concentrations of αCLDN18.2-MMAE for 48 h, apoptosis was assessed. As depicted in Fig. 2A and B, a dose-dependent increase in Annexin V-positive tumor cells was observed, including both early apoptotic (Annexin V^+^/PI^-^) and late apoptotic (Annexin V^+^/PI^+^) cell populations. Additionally, Western Blot was conducted to examine alterations of apoptosis-related protein within MKN45-CLDN18.2 and SNU-601 cells after 48 h of treatment. As illustrated in Fig. 2C and D, elevated expressions of cleaved caspase-9 and cleaved PARP were observed, indicative of the activation of apoptosis-related pathways. These findings collectively confirmed the capacity of αCLDN18.2-MMAE inducing apoptosis in CLDN18.2-positive gastric cancer cells.

### αCLDN18.2-MMAE induced autophagy in CLDN18.2-positive gastric cancer cells

**Fig. 3:**
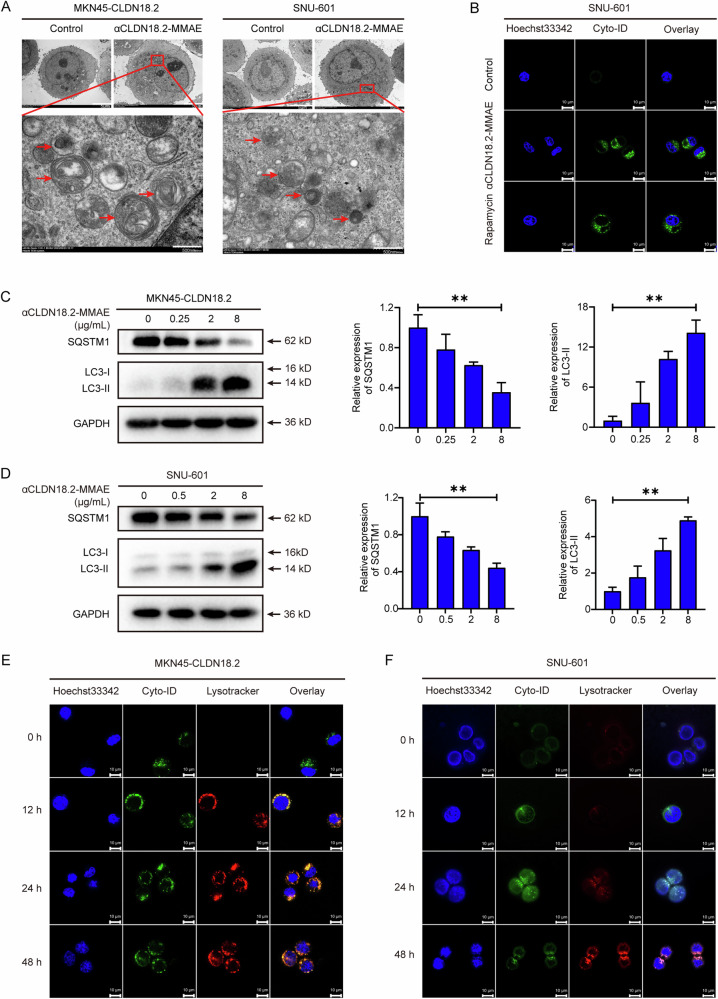
αCLDN18.2-MMAE induced autophagy in CLDN18.2-positive gastric cancer cells. **A** MKN45-CLDN18.2 and SNU-601 cells were exposed to αCLDN18.2-MMAE for 48 h and photographed through transmission electron microscopy. Autophagosomes were marked by red arrows. Scale bar 5 μm (upper) and 500 nm (lower). **B** Cells were treated with αCLDN18.2-MMAE or Rapamycin (positive control). Then, Cyto-ID was utilized to stain autophagosomes (green fluorescence) accumulated in SNU-601 cells, while Hoechst 33342 was employed to locate the cell nucleus (blue fluorescence). Scale bar = 10 μm. **C**, **D** MKN45-CLDN18.2 and SNU-601 cells were subjected to varying concentrations of αCLDN18.2-MMAE. The expression levels of SQSTM1 and LC3 were then detected through Western Blot analysis. The results of repeated experiments were quantified using ImageJ. Data were shown as mean ± S.D. (*n* = 3) and analyzed by ordinary one-way ANOVA (**P < 0.01). **E**, **F** MKN45-CLDN18.2 and SNU-601 cells were treated with αCLDN18.2-MMAE (4 μg/mL) for a specific time, then stained with Hoechst33342, Cyto-ID, and Lysotracker. Hoechst 33342 was utilized to locate cell nuclei, while Cyto-ID and Lysotracker were utilized to identify autophagosomes and lysosomes, respectively. Scale bar = 10 μm.

During the observation of cell morphology through electron microscopy, a significant accumulation of autophagic vesicles was unexpectedly discovered in CLDN18.2-positive gastric cancer cells after αCLDN18.2-MMAE treatment (Fig. 3A). To further validate the induction of autophagy, Cyto-ID, a fluorescent dye specifically labeling autophagosomes, was employed. Similarly, confocal microscopy results showed enhanced green fluorescence within the αCLDN18.2-MMAE-treated cells, akin to the positive control group treated with rapamycin (Figs. 3B and S2). Additionally, Western Blot results indicated an increase in LC3-II expression levels and a decrease in SQSTM1 expression levels (Fig. 3C, D). In autophagy, LC3-I is modified by a ubiquitin-like system and subsequently coupled with phosphatidylethanolamine to form LC3-II. SQSTM1 acts as a bridge between the ubiquitinated proteins and LC3 proteins, ultimately targeted for degradation by autophagy. Therefore, an increase in the ratio of LC3-II to LC3-I expression and a decrease in SQSTM1 expression can indicate the occurrence of autophagy. Furthermore, to observe autophagic flux, Cyto-ID, LysoTracker, and Hoechst 33342 were utilized to stain autophagosomes (green), lysosomes (red), and nuclei (blue), respectively. After a 12-hour treatment with αCLDN18.2-MMAE, notable autophagosomes appeared in both MKN45-CLDN18.2 and SNU-601 cells. Additionally, red fluorescence was also present in MKN45-CLDN18.2 cells, indicative of lysosomal activation. At 24 h, autophagosomes and lysosomes showed bright fluorescence and co-localization, suggesting that autophagosomes fused with lysosomes to form autolysosomes. At 48 h, the green fluorescence was visibly weakened while the red fluorescence remained strong, indicating autophagosome degradation (Fig. 3E, F). In conclusion, αCLDN18.2-MMAE initiated both autophagosome formation and autophagic flux in CLDN18.2-positive gastric cancer cells.

### Inhibiting autophagy reinforced cytotoxicity and apoptosis triggered by αCLDN18.2-MMAE in CLDN18.2-positive gastric cancer cells

**Fig. 4:**
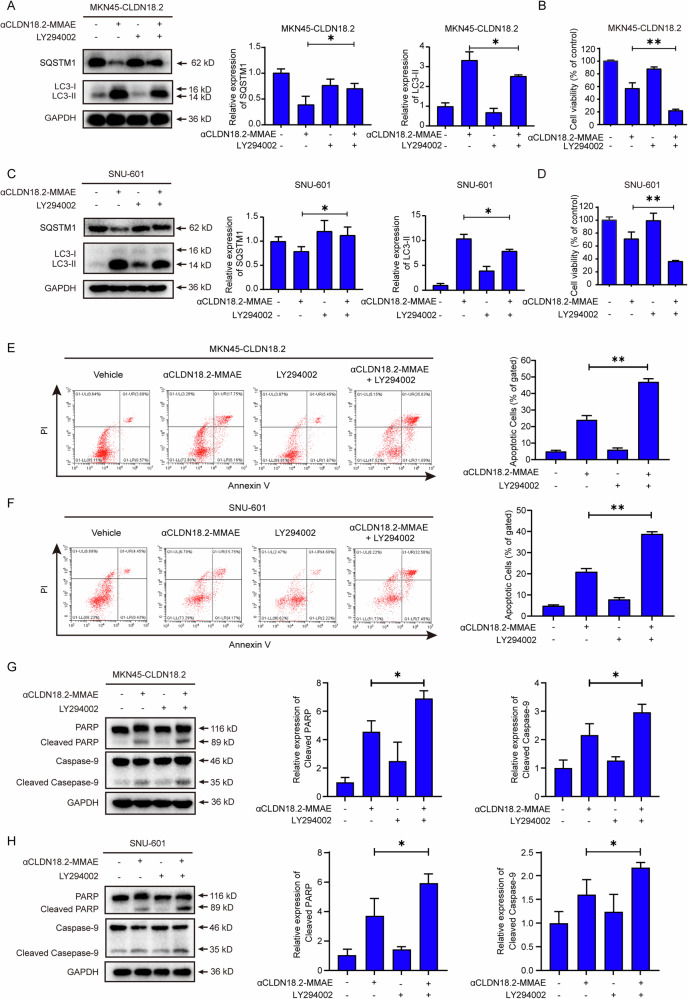
Inhibiting autophagy reinforced cytotoxicity and apoptosis triggered by αCLDN18.2-MMAE in CLDN18.2-positive gastric cancer cells. **A**, **C** The expression levels of SQSTM1 and LC3 were detected and quantified in MKN45-CLDN18.2 and SNU-601 cells after co-treatment with αCLDN18.2-MMAE (4 μg/mL) and LY294002 (0.5 μM). Data were shown as mean ± S.D. (*n* = 3) and analyzed by two-tailed unpaired t-test, *P < 0.05). **B**, **D** After MKN45-CLDN18.2 and SNU-601 cells treated with αCLDN18.2-MMAE (4 μg/mL) and LY294002 (0.5 μM) for 48 h, cell viability was evaluated using CCK-8 assay. Results were shown as mean ± S.D. (*n* = 3) and analyzed by two-tailed unpaired t-test (**P < 0.01). **E**, **F** The percentage of apoptotic cells was determined by flow cytometry after treatment with αCLDN18.2-MMAE (4 μg/mL), with or without LY294002 (0.5 μM). Apoptotic cells were characterized as Annexin V positive cells. The results of three independent experiments were analyzed by two-tailed unpaired t-test (**P < 0.01) and displayed as mean ± S.D. (*n* = 3). **G**, **H** The expression levels of apoptosis-related proteins were detected by Western Blot analyses after indicated cells were treated with αCLDN18.2-MMAE (4 μg/mL) with or without LY294002 (0.5 μM). Data were presented as mean ± S.D. (*n* = 3) and analyzed by two-tailed unpaired t-test (*P < 0.05).

Given the dual role of autophagy in tumor progression, we aimed to investigate whether autophagy inhibition could potentiate the efficacy of αCLDN18.2-MMAE. In comparison to the administration of αCLDN18.2-MMAE alone, co-administration with the autophagy inhibitor LY294002 resulted in a significantly decreased expression of LC3-II protein and an increased expression of SQSTM1, indicating effective autophagy inhibition. (Fig. 4A, C). As shown in Fig. 4B and D, the combination of αCLDN18.2-MMAE with the autophagy inhibitor LY294002 significantly enhanced its cytotoxicity against MKN45-CLDN18.2 and SNU-601 cells. Similarly, co-administration of other autophagy inhibitors such as chloroquine, 3-MA, and hydroxychloroquine also significantly increased the cytotoxic effects of αCLDN18.2-MMAE on these cells (Fig. S3). Furthermore, flow cytometry results showed a substantial augmentation of apoptosis induced by αCLDN18.2-MMAE in MKN45-CLDN18.2 and SNU-601 cells in the combination group (Fig. 4E, F). Similarly, combinational treatment increased the cleavage of PARP and caspase-9 (Fig. 4G, H). Therefore, suppressing αCLDN18.2-MMAE-induced autophagy led to a substantial increase of apoptosis in MKN45-CLDN18.2 and SNU-601 cells. These findings uncovered the cytoprotective role of autophagy in αCLDN18.2-MMAE-treated gastric cancer cells. Combination therapy with autophagy inhibitors may represent a superior approach for eliminating gastric cancer cells compared to αCLDN18.2-MMAE alone.

### Inhibiting autophagy amplified the in vivo antitumor effectiveness of αCLDN18.2-MMAE

**Fig. 5:**
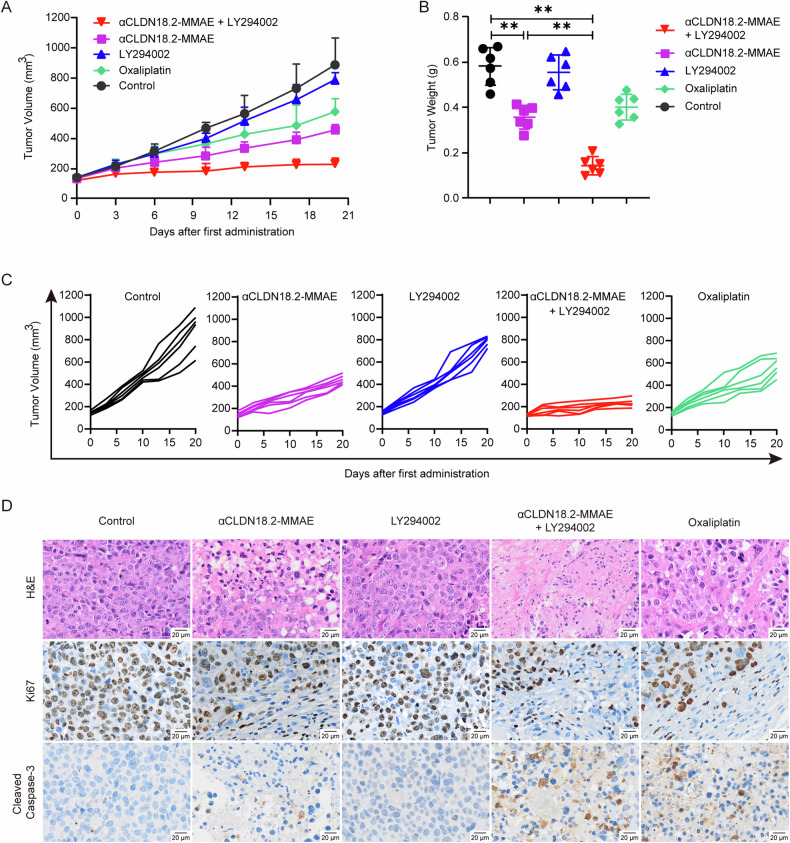
Inhibiting autophagy amplified the in vivo antitumor effectiveness of αCLDN18.2-MMAE. **A**, **C** MKN45-CLDN18.2 cells were subcutaneously implanted in NCG mice to establish xenograft models. αCLDN18.2-MMAE (2 mg/kg), with or without LY294002 (50 mg/kg), was administered twice a week for three weeks. Tumor volume was monitored every three days. Results were presented as mean and error ± S.D. (*n* = 6). **B** At the experimental endpoint, all mice were sacrificed and tumor weight was measured (*n* = 6). Data were analyzed by two-tailed unpaired t-test (**P < 0.01) **D** H&E and immunohistochemical staining of cleaved caspase-3 and Ki-67 in tumor tissues. Scale bar = 20 μm.

After elucidating the role of αCLDN18.2-MMAE-induced autophagy in vitro, we aimed to validate whether inhibiting autophagy can enhance the in vivo antitumor efficacy of αCLDN18.2-MMAE. Subcutaneous xenograft models were established using MKN45-CLDN18.2 cells in NCG mice. During a 21-day treatment of αCLDN18.2-MMAE alone or in combination with LY294002, tumor volume and tumor weight were assessed (Fig. 5A–C). LY294002 did not show any antitumor effects in the MKN45-CLDN18.2 xenograft model when compared with negative control. Remarkably, in the mice treated with combination therapy, tumor growth was significantly inhibited compared to other groups, with the tumor inhibitory rate for combination therapy reaching 74.2%, αCLDN18.2-MMAE alone at 48.8%, and oxaliplatin at 35.9%. H&E staining revealed increased necrosis in tumor tissue following combination treatment of αCLDN18.2-MMAE and LY294002 (Fig. 5D). Additionally, the combination group displayed heightened expression of cleaved caspase-3 and reduced expression of Ki-67 expression in tumor tissues, indicating an increase in apoptosis and a decrease in proliferation (Fig. 5D). Moreover, H&E staining of heart, liver, spleen, lung, kidney and brain tissues from mice in the experimental group showed no obvious morphological damage caused by drugs compared to the control group (Fig. S4). In conclusion, the in vivo antitumor activity of αCLDN18.2-MMAE was further enhanced by inhibiting autophagy with LY294002, evidenced by augmented apoptosis.

### The Akt/mTOR signaling pathway was implicated in the induction of autophagy by αCLDN18.2-MMAE

**Fig. 6:**
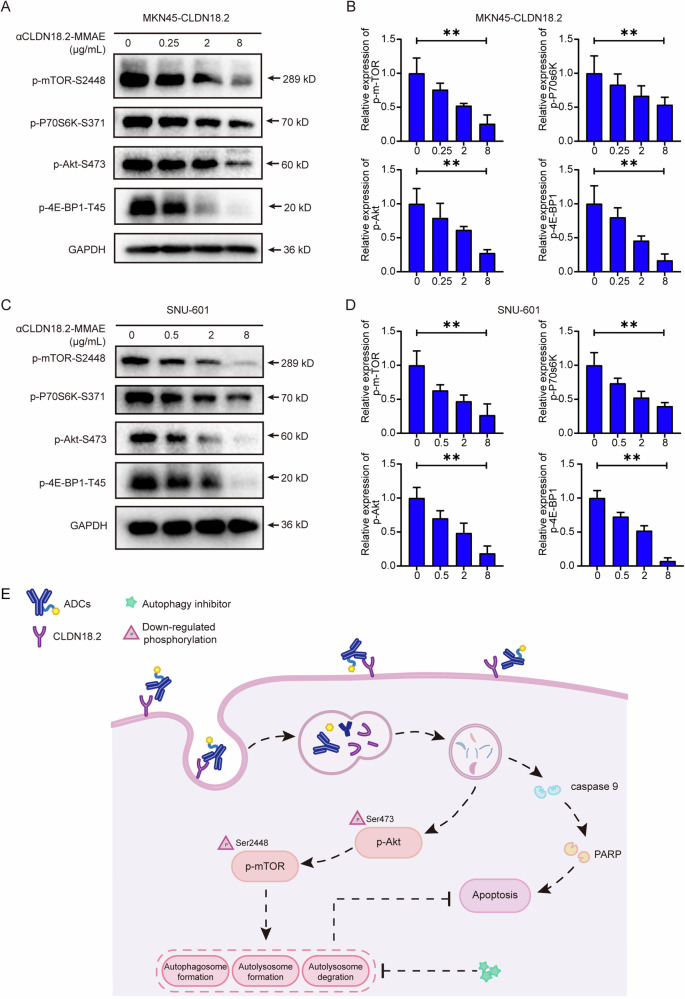
The Akt-mTOR signaling pathway was implicated in the induction of autophagy by αCLDN18.2-MMAE. **A**, **C** MKN45-CLDN18.2 and SNU-601 cells were cocultured with different concentrations of αCLDN18.2-MMAE for 48 h. Western Blot analyses were performed to evaluate the phosphorylation levels of mTOR, P70S6K, Akt, 4E-BP1. **B**, **D** The grayscale intensities of protein bands in A and C were quantified and presented as mean ± S.D. (*n* = 3). Data were analyzed by ordinary one-way ANOVA (**P < 0.01). **E** A Schematic of αCLDN18.2-MMAE targeting CLDN18.2-positive gastric cancer cells for cytotoxicity and autophagy induction. Initially, αCLDN18.2-MMAE binds to CLDN18.2-positive gastric cancer cells, undergoes endocytosis, and releases MMAE. This process initiates caspase-9/PARP-dependent apoptosis while simultaneously inducing cytoprotective autophagy. Co-administration of autophagy inhibitors effectively suppresses protective autophagy in tumor cells, thereby enhancing the antitumor effect of αCLDN18.2-MMAE.

To explore the mechanisms underlying autophagy, we analyzed the alterations in autophagy-related signaling pathways, focusing on changes in the expression levels of key components to elucidate the intrinsic mechanisms of αCLDN18.2-MMAE-induced autophagy. The Western Blot results demonstrated a dose-dependent reduction in phosphorylated mTOR in response to αCLDN18.2-MMAE. Simultaneously, the expression levels of phosphorylated Akt, an upstream signaling protein of mTOR, exhibited a dose-dependent decrease. Furthermore, downstream effectors of Akt/mTOR, including 4E-BP1 and p70S6K, were markedly inhibited (Fig. 6A–D). Given that inhibition of this pathway has been reported to be associated with autophagy activation, these results provided insights into the mechanism underlying autophagy activation induced by αCLDN18.2-MMAE (Fig. 6E).

## Discussion

The primary treatment for gastric cancer is surgery and systemic chemotherapy. Unfortunately, the overall prognosis for patients remains poor. To address this challenge, targeted therapies against HER2, CLDN18.2, EGFR, etc., have been developed, with many showing satisfactory results. For example, HER2 is overexpressed in about 20% of gastric cancers. HER2-targeting trastuzumab deruxtecan was approved to treat patients with HER2-positive unresectable or metastatic gastric cancer [2]. CLDN18.2 is a highly selective biomarker with low expression in normal tissues and high expression in a broader population suffering from digestive malignancies. In a phase III trial of 2104 patients with locally advanced or metastatic gastric adenocarcinoma, 38.4% of the tumors were determined to be CLDN18.2 positive [25]. Furthermore, the successful phase III clinical trial of zolbetuximab has proven the efficacy and safety of CLDN18.2 as a potential therapeutic target for gastric cancer. Additionally, CLDN18.2 has demonstrated promising outcomes in ADC therapies, particularly the rapid advancements of IBI343. IBI343 was designed for the treatment of HER2-negative but CLDN18.2-positive gastric cancer and has progressed to a pioneering phase III clinical trial. Despite research into the efficacy of several ADCs targeting CLDN18.2, little headway has been made in elucidating their mechanisms in gastric cancer, underscoring the need and importance of the present study.

Autophagy is a metabolic process that maintains the stability of the intracellular environment and adapts to changes in the external environment. Despite extensive research, the anti-tumor or pro-tumor role of autophagy in gastric cancer remains controversial [26, 27]. For example, a study reported that the combination of adriamycin and Tanshinone IIA induced autophagy, promoted cell apoptosis, and boosted gastric cancer cell sensitivity to adriamycin [28]. On the other hand, activation of autophagy can also yield opposite results. Chemotherapy drugs like 5-fluorouracil and cisplatin were reported to induce cytoprotective autophagy in gastric cancer [29–31]. As far as we know, there has been no study investigating whether ADCs targeting CLDN18.2 induce autophagy and the potential role of autophagy in ADC therapy for gastric cancer. Our study for the first time reveals that CLDN18.2-targeted ADC can trigger cytoprotective autophagy in gastric cancer, filling this knowledge gap. While exploring the effect of αCLDN18.2-MMAE on gastric cancer treatment, we observed that MKN45-CLDN18.2 and SNU-601 cells formed autophagosomes after treatment with αCLDN18.2-MMAE. Subsequent validation via immunoblotting to evaluate alternations in autophagy-related protein expression, and immunofluorescence to monitor autophagic flux confirmed the induction of autophagy by αCLDN18.2-MMAE. Moreover, our findings indicated that αCLDN18.2-MMAE-induced autophagy served a cytoprotective role, co-administration of autophagy inhibitor enhanced the antitumor effect of αCLDN18.2-MMAE, as evidenced by increased induction of tumor cell apoptosis and enhanced suppression of tumor growth.

To probe into the mechanism behind αCLDN18.2-MMAE-induced autophagy, we examined various autophagy-related signaling pathways. Specifically, treatment of CLDN18.2-positive gastric cancer cells with αCLDN18.2-MMAE led to a decreased phosphorylation of Akt, mTOR, and two critical downstream effectors, 4E-BP1 and p70S6K [32], indicating the inactivation of the Akt/mTOR pathway. The Akt/mTOR signaling cascade is a classic driver pathway in human cancers [33, 34]. Once Akt is phosphorylated and activated, it can directly target tuberous sclerosis complex 2 (TSC2), a component of the TSC1/2, leading to its inactivation. Consequently, TSC2 loses its inhibitory effect on the Ras homolog enriched in brain (Rheb) protein. Activated Rheb protein binds to mTOR complex 1 (mTORC1), promoting the activation of the mTORC1 signaling pathway [35]. Recent research findings have revealed that mTORC1 inhibition not only initiates autophagy but also directly regulates various autophagic processes, including nucleation, elongation, maturation, and termination of autophagosomes [36]. Our research further confirmed the involvement of the Akt/mTOR pathway in autophagy induction by αCLDN18.2-MMAE.

In conclusion, our study demonstrated the antitumor efficacy of αCLDN18.2-MMAE against CLDN18.2-positive gastric cancer. Importantly, we reported for the first time that CLDN18.2-directed ADCs induced cytoprotective autophagy in gastric cancer treatment, with involvement of the Akt/mTOR signaling pathway. Our findings provided theoretical support and experimental evidence for the potential of CLDN18.2-directed ADCs, in combination with autophagy inhibitors, to enhance therapeutic efficacy in CLDN18.2-positive gastric cancer.

## Methods and materials

### Cell culture

Human gastric cancer cell lines MKN45 and SNU-601 were obtained from Nanjing COBIOER Bioscience Co., Ltd. MKN45-CLDN18.2, a stable transduced cell line overexpressing CLDN18.2, was provided by Shanghai Junshi Biosciences Co., Ltd. Human normal gastric mucosal epithelial cell line GES-1 was obtained from Dr. Xiao-Jing Shi at Zhengzhou University. SNU-601 and GES-1 cells were cultured in the RPMI1640 medium, while MKN45 and MKN45-CLDN18.2 cells were cultured in DMEM. Cells were incubated at 37 °C in a 5% CO_2_ atmosphere to ensure optimal growth conditions and experimental consistency. All cell lines were verified as mycoplasma-free using the Myco-Lumi™ Luminescent Mycoplasma Detection Kit (Beyotime, Shanghai, China, C0297S).

### Cell viability assay

Cells were seeded at a density of approximately 5000 cells per well in 96-well plates. After 24 h, the cells were co-incubated with the tested compound for 48 h. The original culture medium was then removed, and each well was replenished with a medium containing 10% Cell Counting Kit-8 (CCK-8) (Beyotime, C0038, Shanghai, China). The cells were then incubated at 37 °C for 1.5 h. After incubation, viable cell absorbance was assessed at 450 nm with a microplate reader.

### Transmission electron microscopy analysis

After being treated with or without αCLDN18.2-MMAE (4 μg/mL) for 48 h, MKN45-CLDN18.2 and SNU-601 cells were gently collected and subsequently fixed in electron microscopy buffer. The fixed cells were then post-fixed in 1% osmium tetroxide for 4 h, rinsed 3 times by phosphate buffer, and dehydrated under gradient ethanol series. Next, the samples were embedded in epoxy resin. Then these resin blocks were sliced, stained, and examined using a transmission electron microscope (Hitachi, HT7800).

### Confocal microscopy

To investigate autophagosomes, MKN45-CLDN18.2 and SNU-601 cells were separately seeded onto confocal dishes. Following treatment with αCLDN18.2-MMAE (4 μg/mL) or rapamycin (50 nM) for 24 h, Hoechst 33342 (Meilunbio, MA0126, Dalian China) and Cyto-ID (ENZO Life Science, ENZ-51031-K200, NY US) were added and incubated for thirty minutes at 37 °C. Fluorescent images were acquired using a fluorescence microscope (Carl Zeiss LSM710). To evaluate autophagic flux, MKN45-CLDN18.2 and SNU-601 cells were seeded onto separate confocal dishes. After 24 h, treatment with αCLDN18.2-MMAE (4 μg/mL) was initiated. At 0, 12, 24, and 48 h post-treatment, cells were exposed to serum-free culture medium containing CYTO-ID® Green Detection Reagent, LysoTracker™ Red DND-99 (Thermo Fisher, L7528, MA US), and Hoechst 33342, as per the manufacturer’s instructions. Following a 30-minute incubation, cellular observations were conducted using a confocal microscope.

### Flow cytometry

To assess CLDN18.2 expression on the cell surface, cells were harvested and categorized into anti-CLDN18.2 antibody and isotype groups. The anti-CLDN18.2 antibody group was treated with the primary antibody (DIMA Biotech, DME100179, Wuhan China) and incubated at 4 °C for 30 minutes. After dual PBS washes, a secondary antibody (ABclonal, AS056, Wuhan China) was added, followed by a 30-minute incubation at 4 °C in the dark. After two washes with PBS, cells were resuspended for the next analysis. For analyzing cell apoptosis, MKN45-CLDN18.2 and SNU-601 cells were treated with gradient concentrations of αCLDN18.2-MMAE, and Annexin V^+^/PI^+^ or Annexin V^+^/PI^−^ cells were detected and classified as apoptotic cells.

### Western Blot analysis

Total proteins from MKN45-CLDN18.2 and SNU-601 cells were extracted using RIPA lysis buffer (Beyotime, P0013D, Shanghai China). Equal amounts of protein from each sample underwent SDS-PAGE and were transferred to PVDF membranes. After incubation with 5% BSA, membranes were probed with primary antibodies specific for PARP (Cell Signaling Technology, 9532, MA US), caspase-9 (Cell Signaling Technology, 9502, MA US), SQSTM1 (Proteintech, 18420-1-AP, Wuhan China), LC3A/B (Cell Signaling Technology, 12741S, MA US), p-mTOR-S2448 (Proteintech, 67778-1-Ig, Wuhan China), p-P70S6K-S371 (Cell Signaling Technology, 9208, MA US), p-Akt-S473 (Cell Signaling Technology, 4060S, MA US), p-4E-BP1-T45 (Cell Signaling Technology, 2855S, MA US), and GAPDH (Servicebio, GB15002-100, Wuhan China). After rigorous washing with TBST, the membranes underwent a 2-hour incubation with peroxidase-conjugated secondary antibodies. Immunoreactive bands were visualized using ECL Luminescence Reagent (Meilunbio, MA0186, Dalian China).

### Tumor xenograft model

All animal experiments strictly adhered to the guidelines and protocols approved by the Animal Ethical Committee of School of Pharmacy Fudan University. MKN45-CLDN18.2 cells were harvested, suspended in PBS, and subcutaneously implanted at a density of 5 × 10^6 cells per NCG mouse. Randomization occurred when the tumor volume reached around 100 mm^3. To assess the antitumor efficacy of αCLDN18.2-MMAE, mice were allocated into three groups receiving PBS, αCLDN18.2-MMAE (2 mg/kg), or oxaliplatin (5 mg/kg) twice weekly. Regular measurements of tumor dimensions (length and width) were taken. To investigate whether co-administration of the autophagy inhibitor LY294002 enhances the antitumor effects of αCLDN18.2-MMAE, mice were randomly assigned to four groups and received intraperitoneal injections of PBS, αCLDN18.2-MMAE (2 mg/kg), LY294002 (50 mg/kg), or a combination of αCLDN18.2-MMAE and LY294002, twice weekly for 3 weeks. Periodic measurements of tumor dimensions were conducted, and tumor volume was calculated using the formula 1/2 × (long diameter in mm) × (short diameter in mm) × (short diameter in mm). Tumor growth inhibition rate (%) = (1 – (mean volume of treated tumors) / (mean volume of control tumors)) × 100%.

### Hematoxylin and Eosin (H&E) staining

Following the acquisition of cellular sections, paraffin removal from the sections was carried out using xylene, followed by a series of alcohol solutions in descending concentrations and ultimately immersion in distilled water. Subsequently, the sections underwent staining with hematoxylin and eosin. Dehydration of the stained sections was achieved with absolute ethanol, followed by rendering the sections transparent with xylene. The processed sections were then mounted, and observations were made under a microscope.

### Statistical analysis

The significance of the difference was figured out by Student’s *t*-test or one-way ANOVA with GraphPad Prism 9.0.0. When the *P* value is more than or equal to 0.05, differences were considered to be not significant and marked as “ns”. When the *P* value is less than 0.05, differences were considered to be statistically different and marked as “*”. When the *P* value is less than 0.01, differences were marked as “**”. The results of several independent experiments were displayed as mean ± standard deviations (S.D.).

## Supplementary information

Supplemental Material

Supplementary Figure

## Data Availability

The data and materials are available by contacting the corresponding author upon reasonable request.
