# Supplementary material for: Enhancing antitumor efficacy of CLDN18.2-directed antibody-drug conjugates through autophagy inhibition in gastric cancer
Source: Cell Death Discov. 2024 Sep 3;10:393. doi: 10.1038/s41420-024-02167-0 (PMC11372199; doi:10.1038/s41420-024-02167-0)

**Supplementary information**

**Western Blot**

**Enhancing Antitumor Efficacy of CLDN18.2-directed antibody-drug conjugates through Autophagy Inhibition in Gastric Cancer**

Wenjing Xue^1#^, Caili Xu^1#^, Kaiqi Zhang^2#^, Lu Cui^2^, Xiting Huang^1^, Yanyang Nan^1^, Dianwen Ju^1^*, Xusheng Chang^2^*, Xuyao Zhang^1^*

^1^Department of Biological Medicines & Shanghai Engineering Research Center of Immunotherapeutics, School of Pharmacy, Fudan University, Shanghai, 201203, China

^2^Department of Gastrointestinal Surgery, Changhai Hospital, Naval Medical University, Shanghai 200433, China

^#^These authors contributed equally to this work.

**Running title:** αCLDN18.2-MMAE and autophagy in gastric cancer

***Corresponding Author:**

Dianwen Ju, [dianwenju@fudan.edu.cn](mailto:dianwenju@fudan.edu.cn)

Xusheng Chang, [cxs20051014@163.com](mailto:cxs20051014@163.com)

Xuyao Zhang, [xuyaozhang@fudan.edu.cn](mailto:xuyaozhang@fudan.edu.cn)

**Supplementary material1:**

**Figure 2C**

MKN45-CLDN18.2

PARP and Cleaved PARP
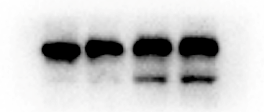

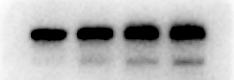

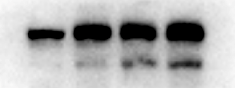


Caspase-9 and Cleaved caspase-9
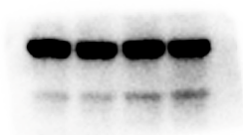

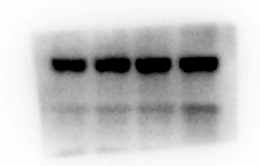

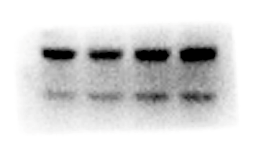


GAPDH
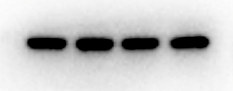

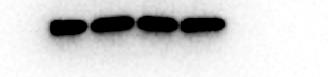

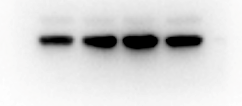


**Supplementary material2:**

**Figure 2D**

SNU-601

PARP and Cleaved PARP
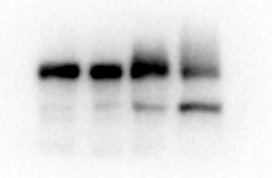

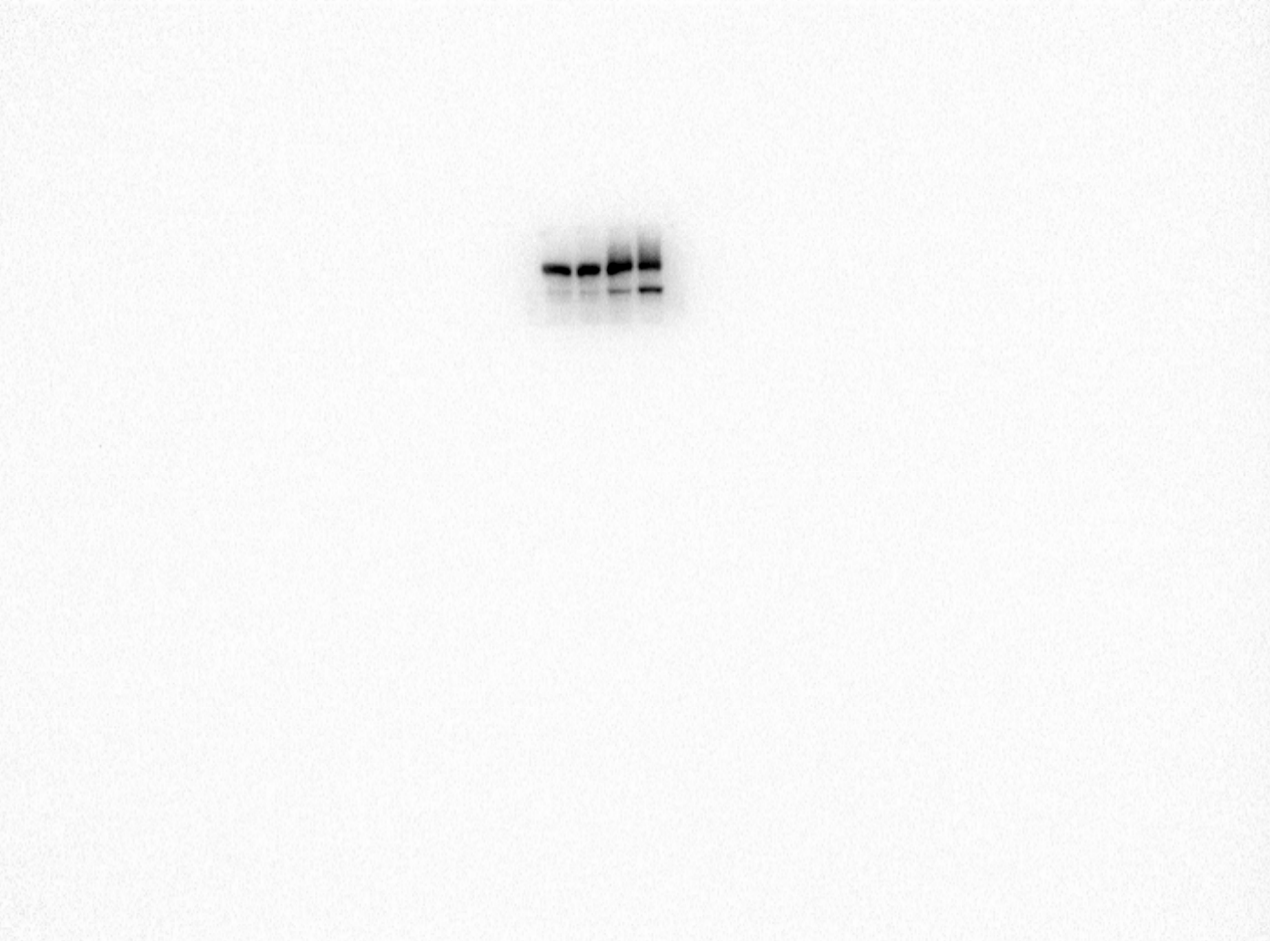

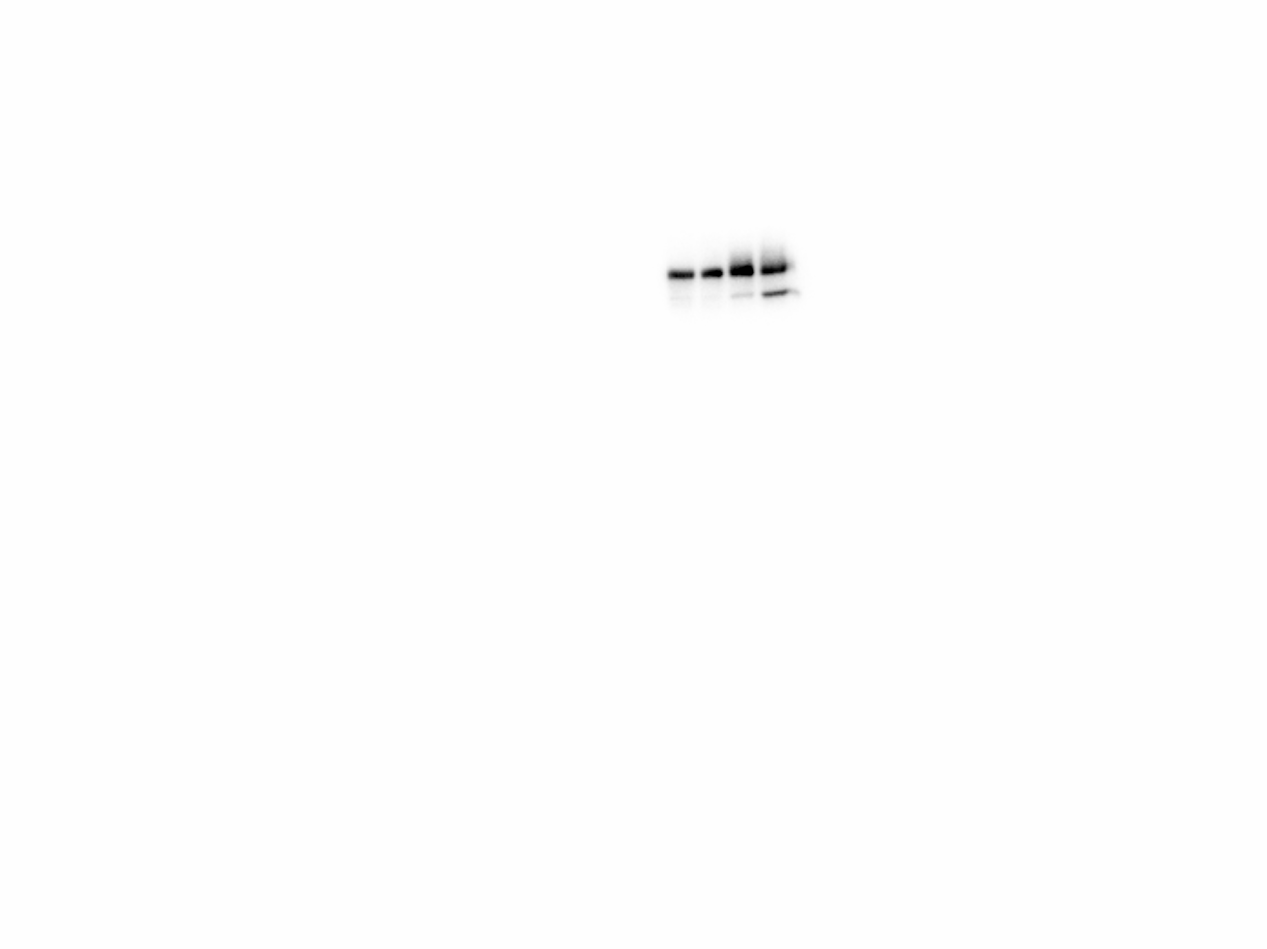


Caspase-9 and Cleaved caspase-9
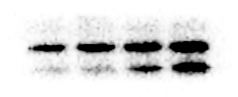

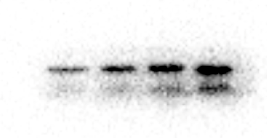

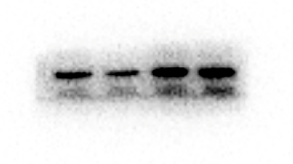


GAPDH
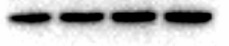

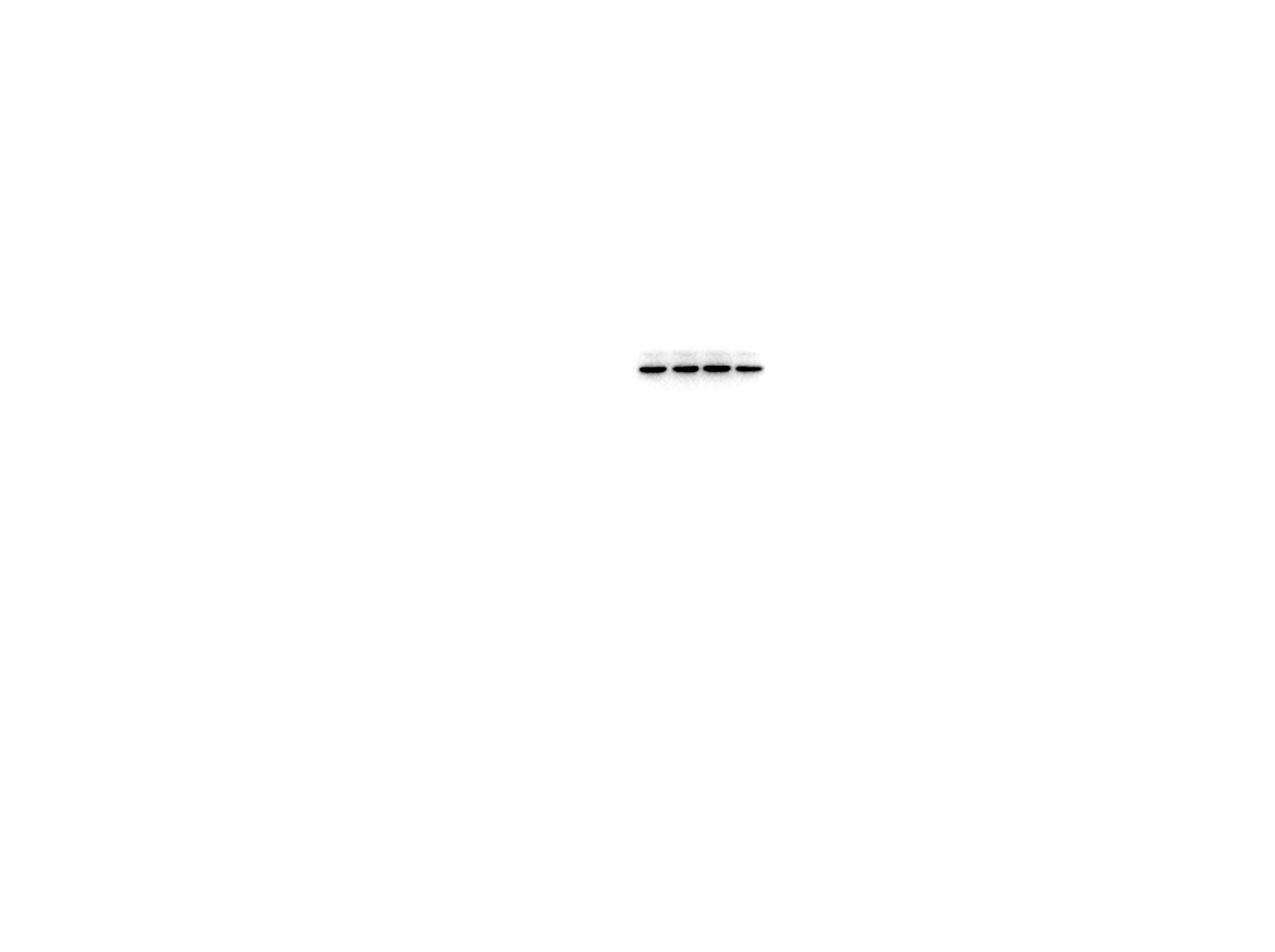

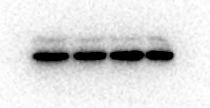


**Supplementary material3:**

MKN45-CLDN18.2

SQSTM1
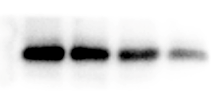

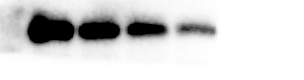

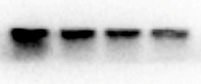


LC3-I and LC3-II
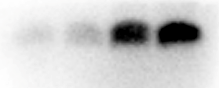

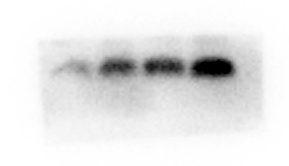

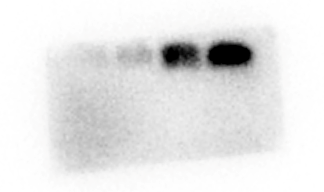


GAPDH
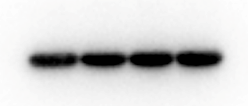

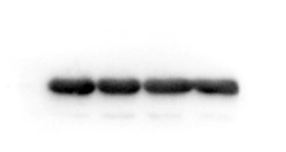

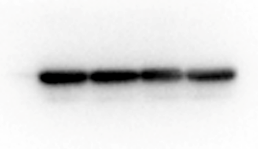


**Supplementary material4:**

**Figure 3D**

SNU-601

SQSTM1
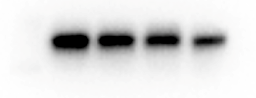

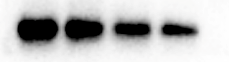

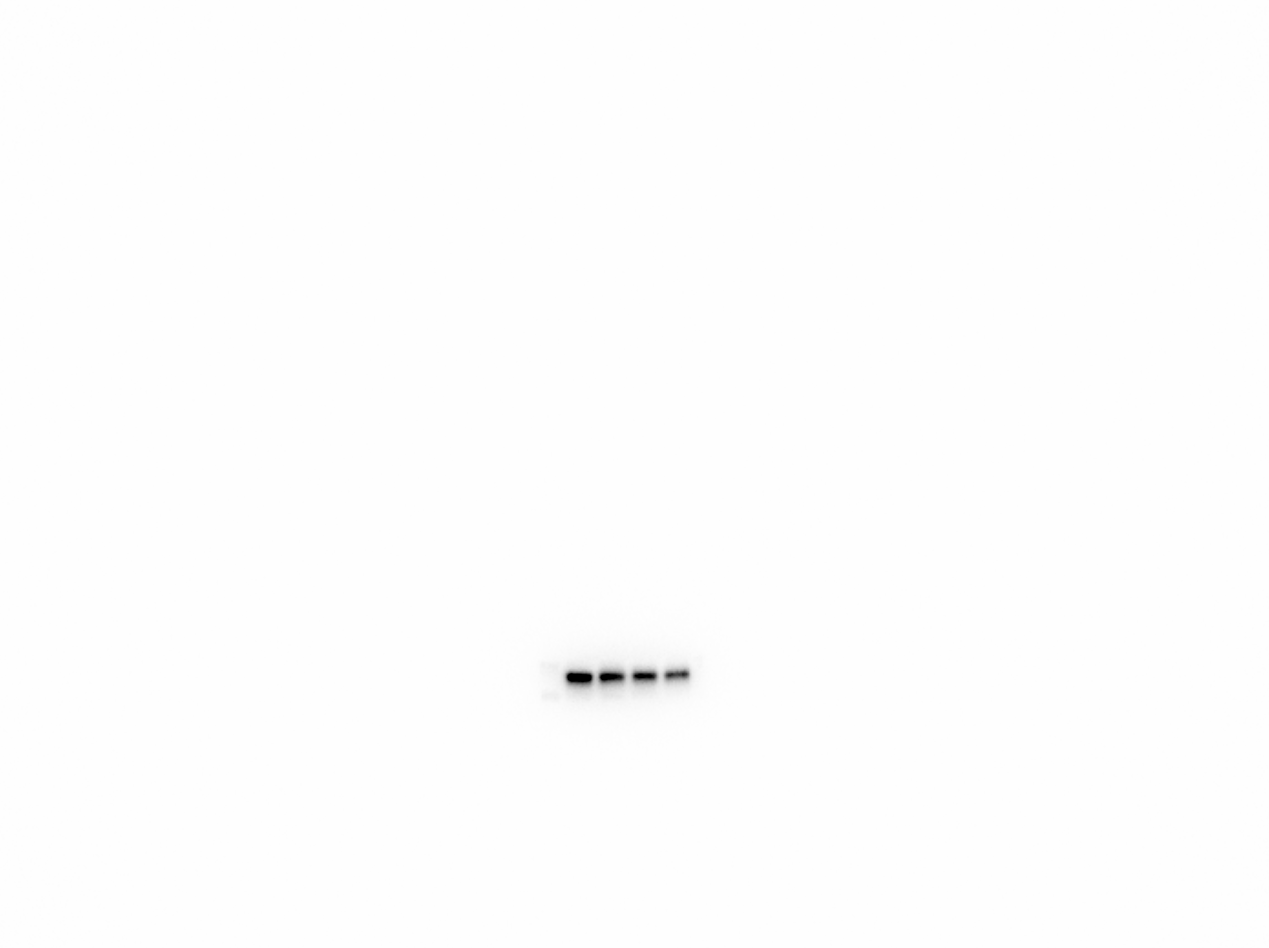


LC3-I and LC3-II
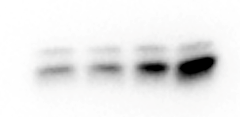

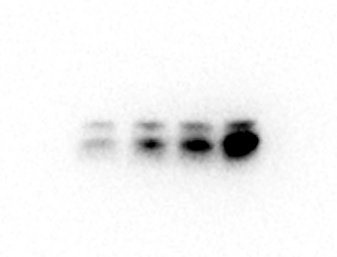

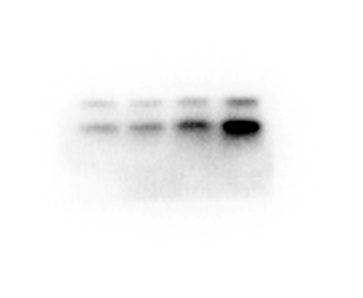


GAPDH
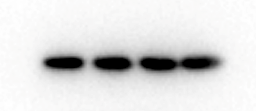

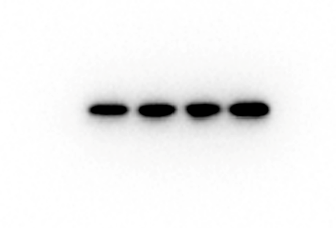

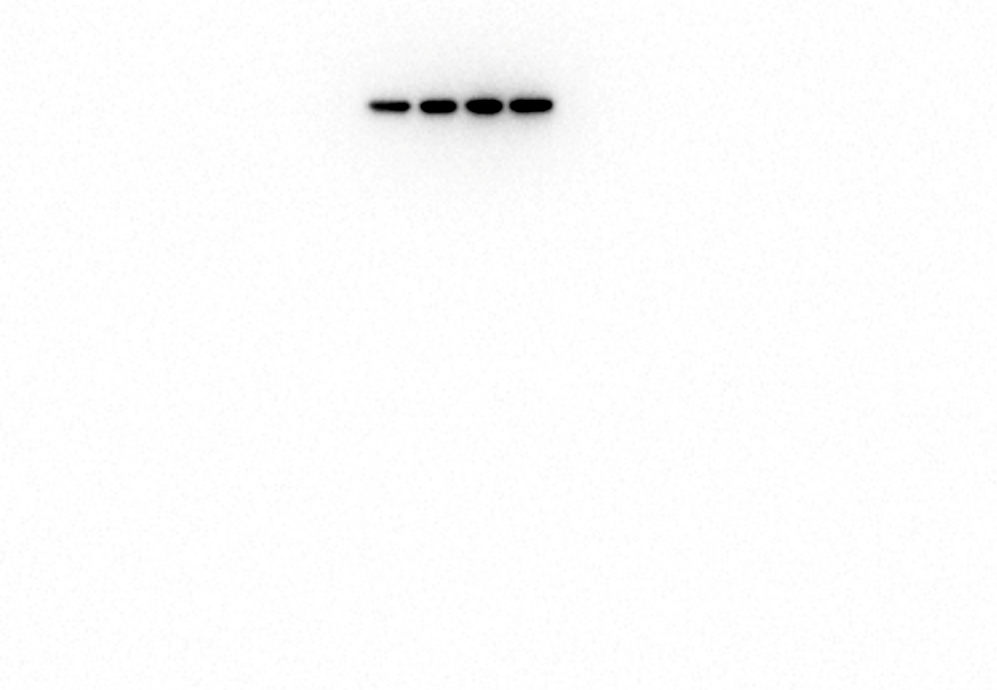


**Supplementary material5:**

**Figure 4A**

MKN45-CLDN18.2

SQSTM1
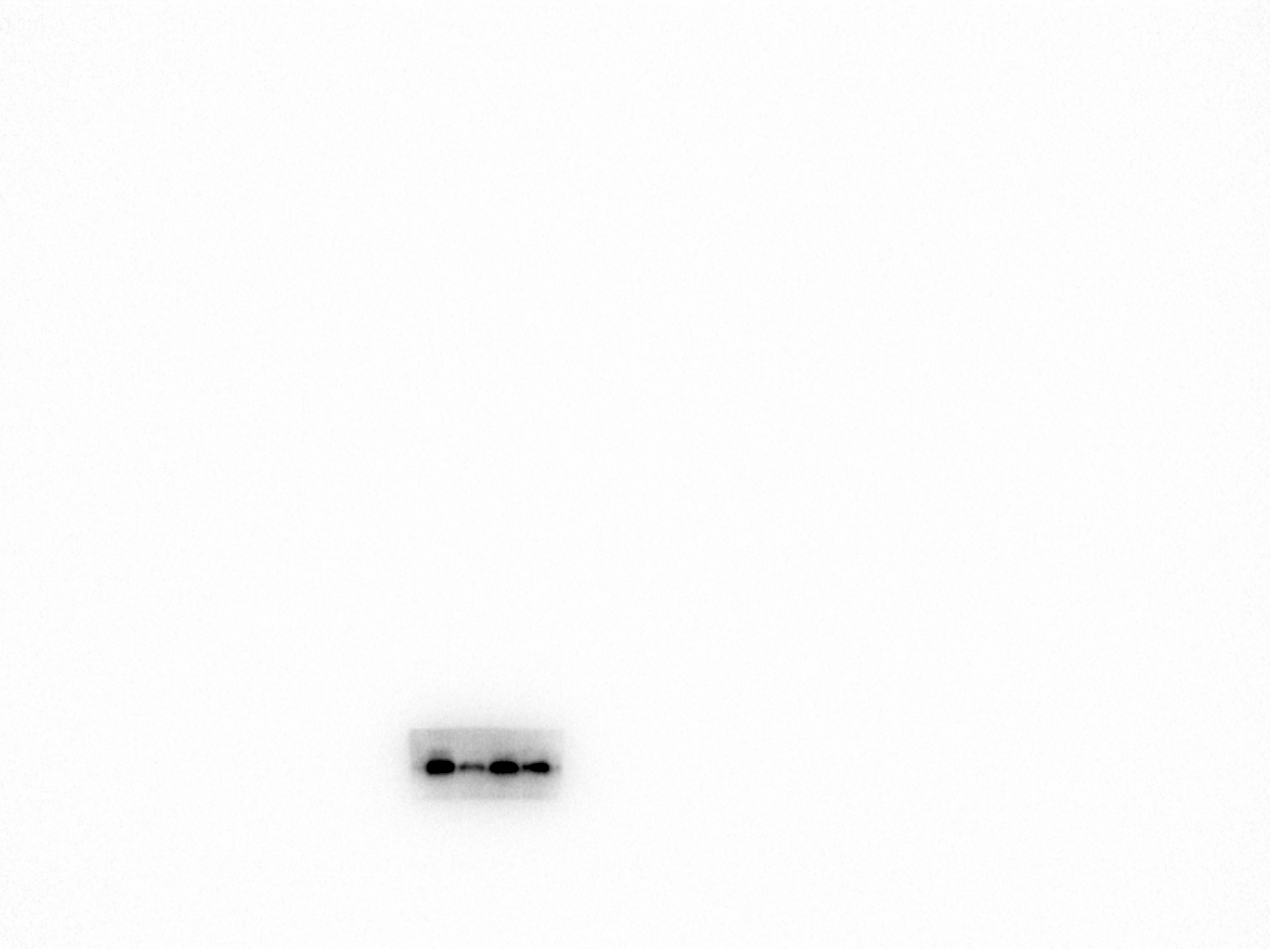

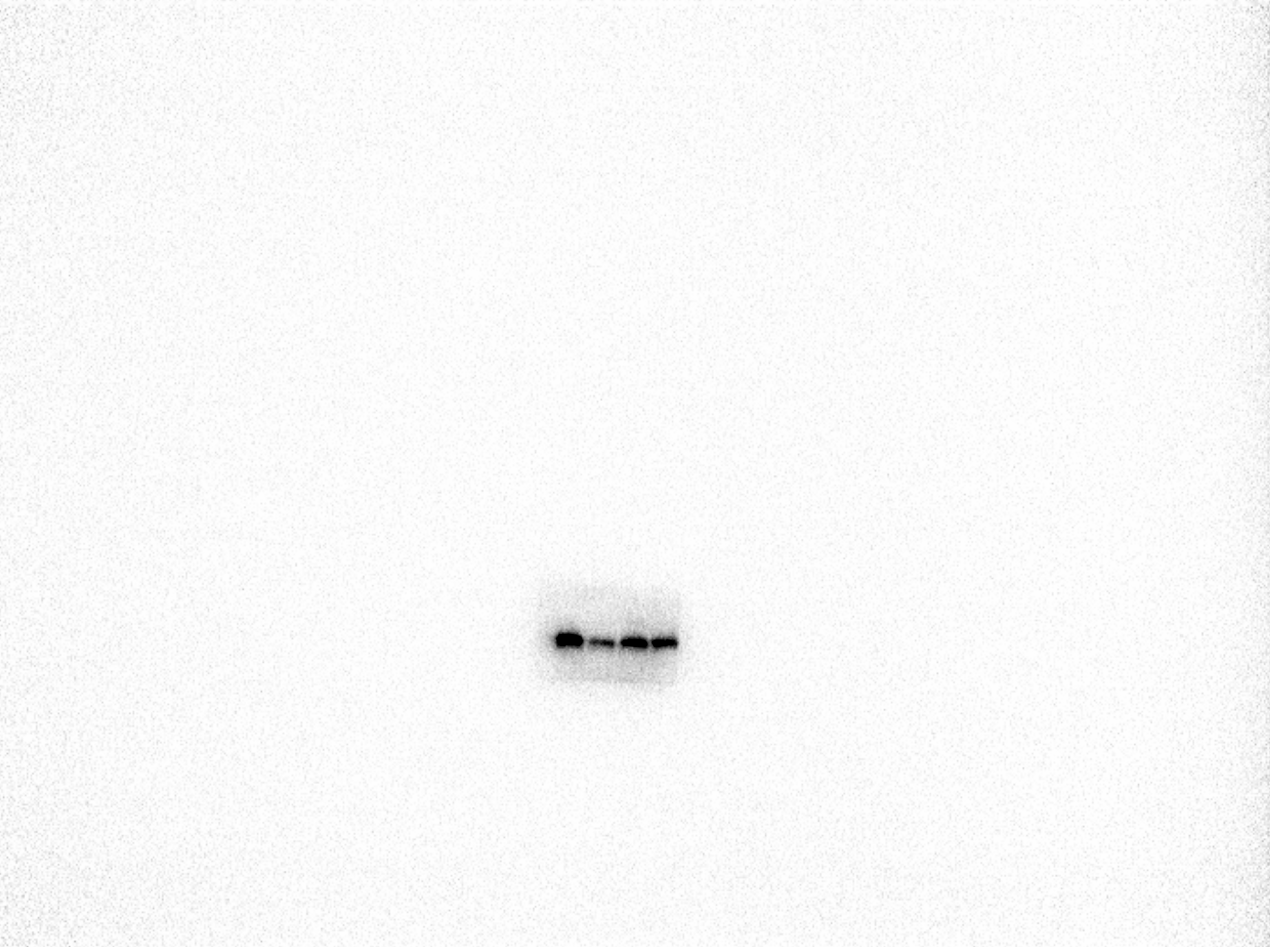

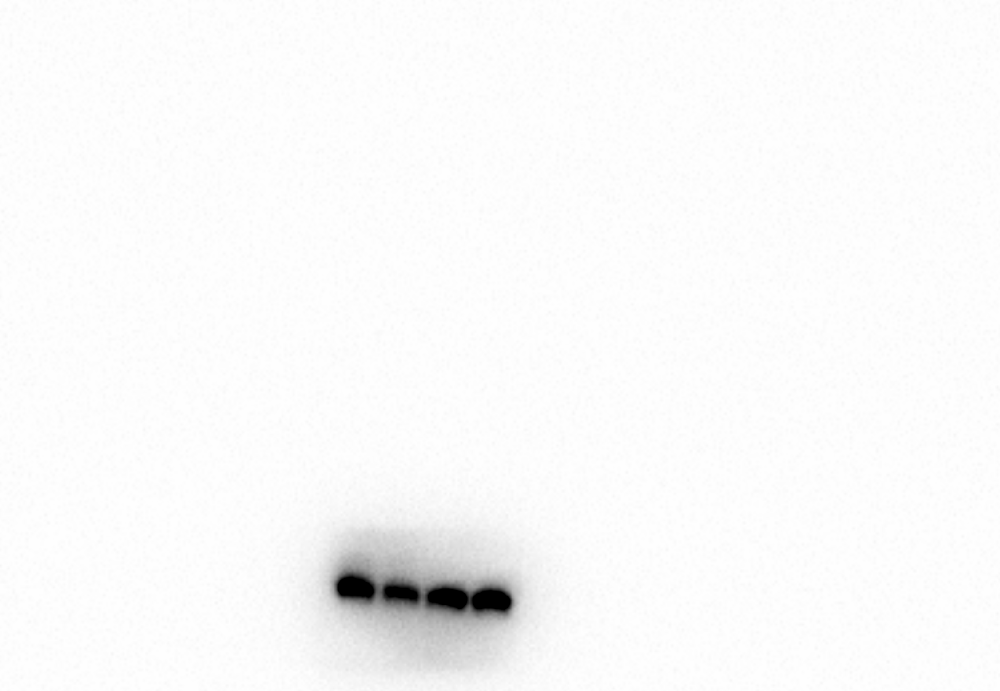


LC3-I and LC3-II
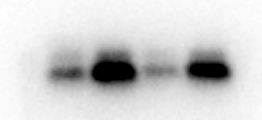

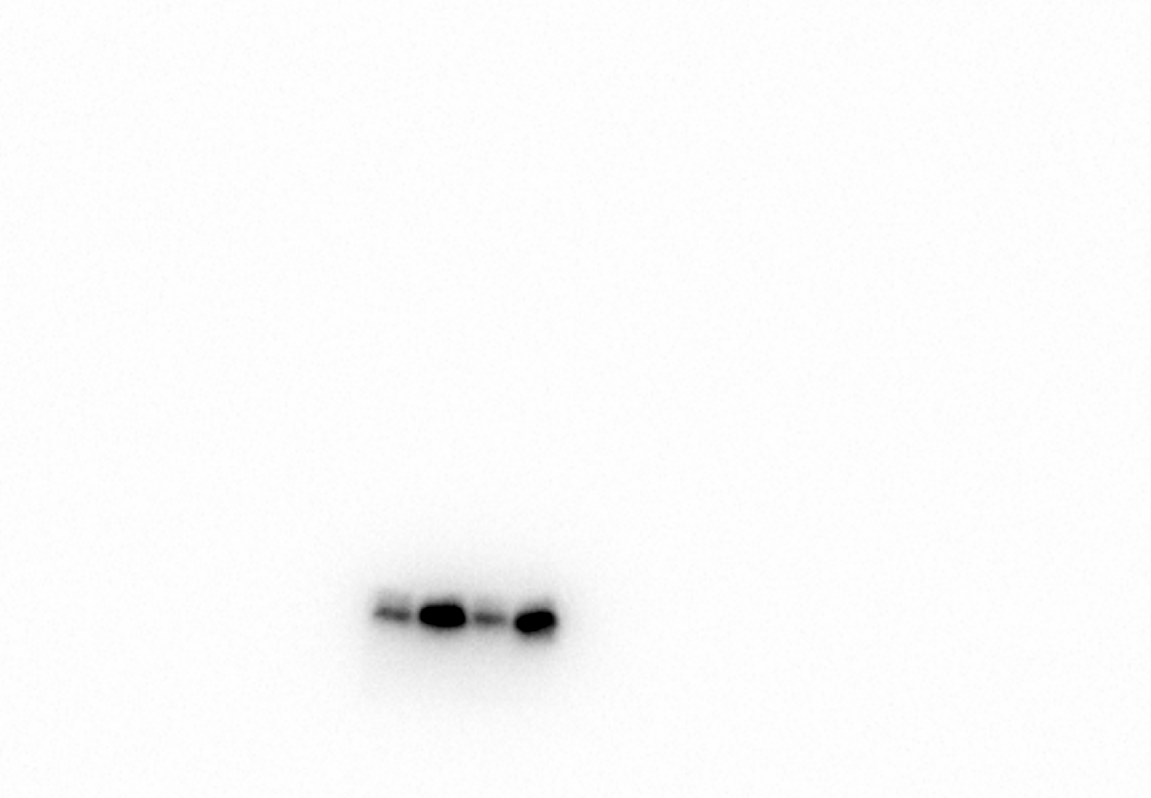

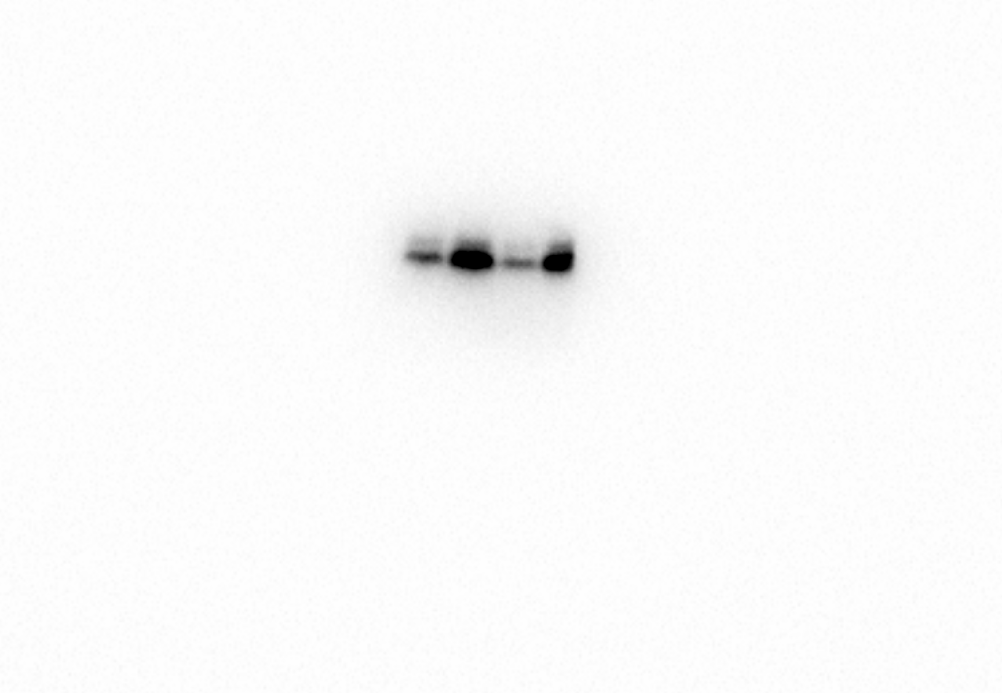


GAPDH
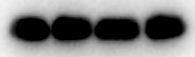

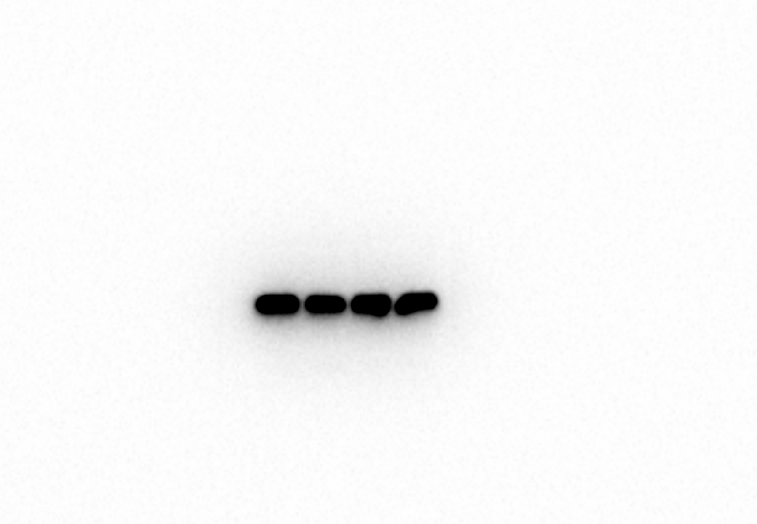

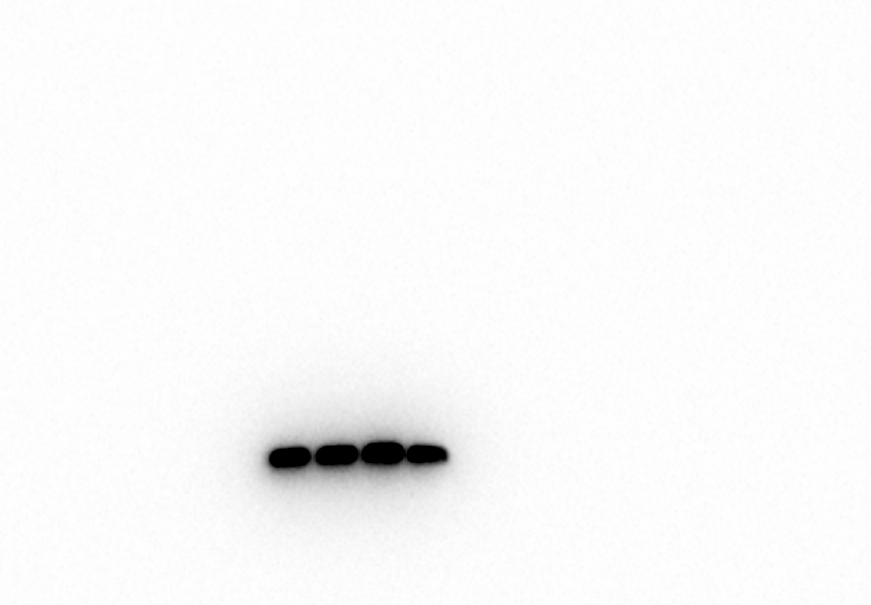


**Supplementary material6:**

**Figure 4C**

SNU-601

SQSTM1
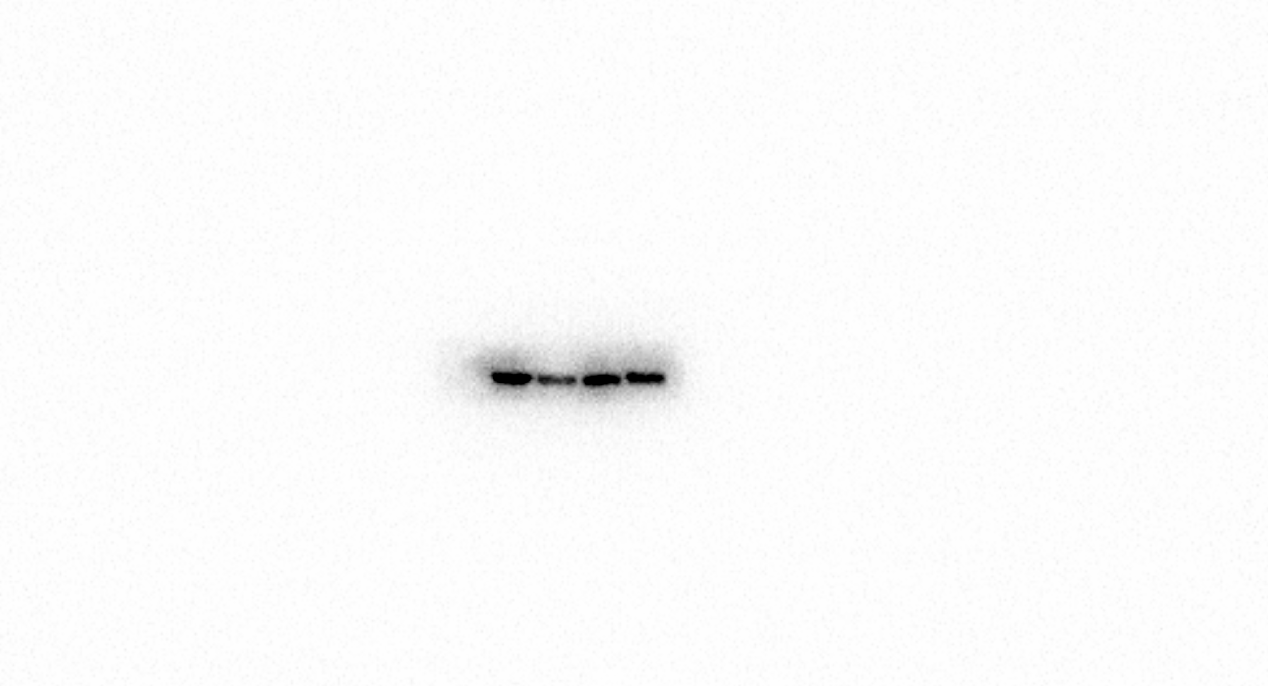

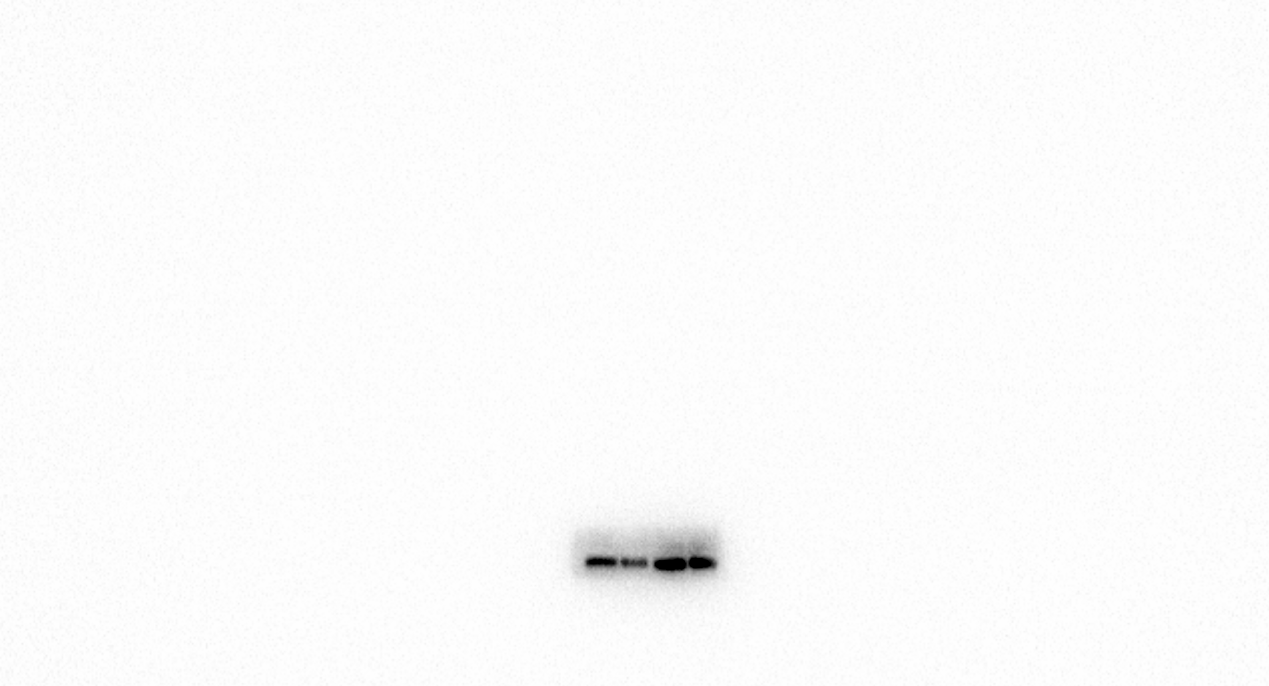

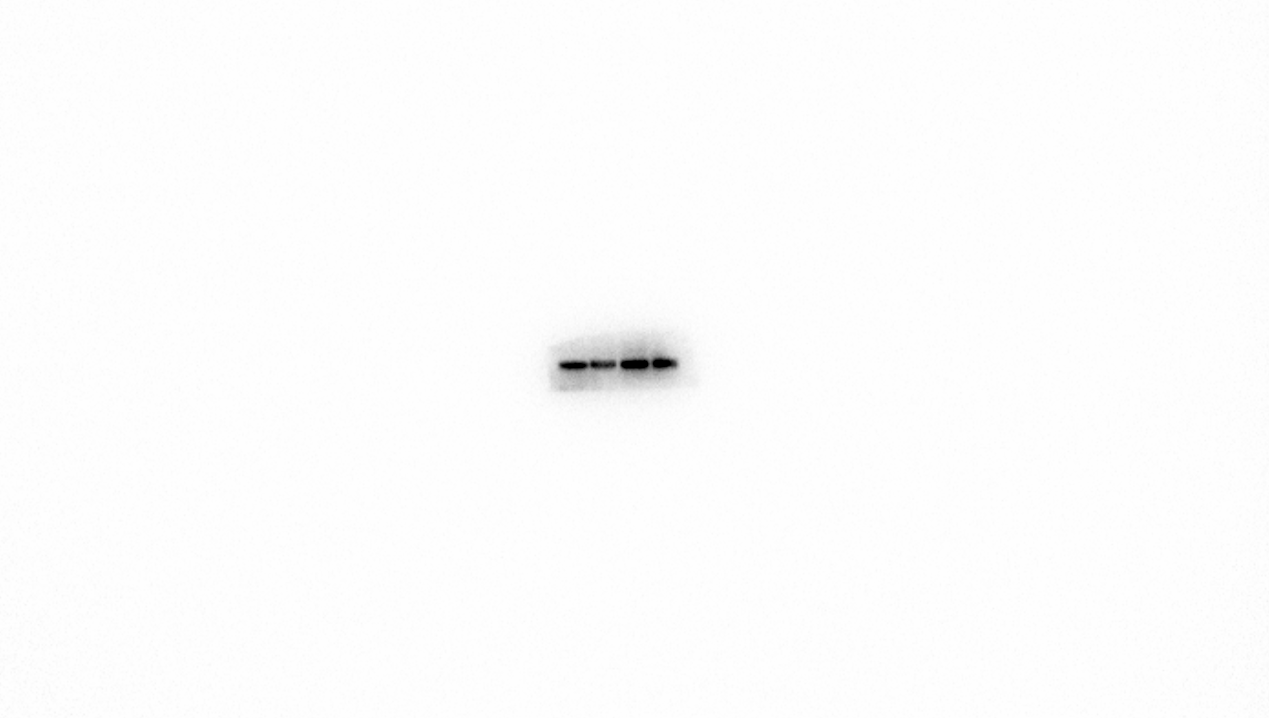


LC3-I and LC3-II
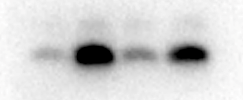

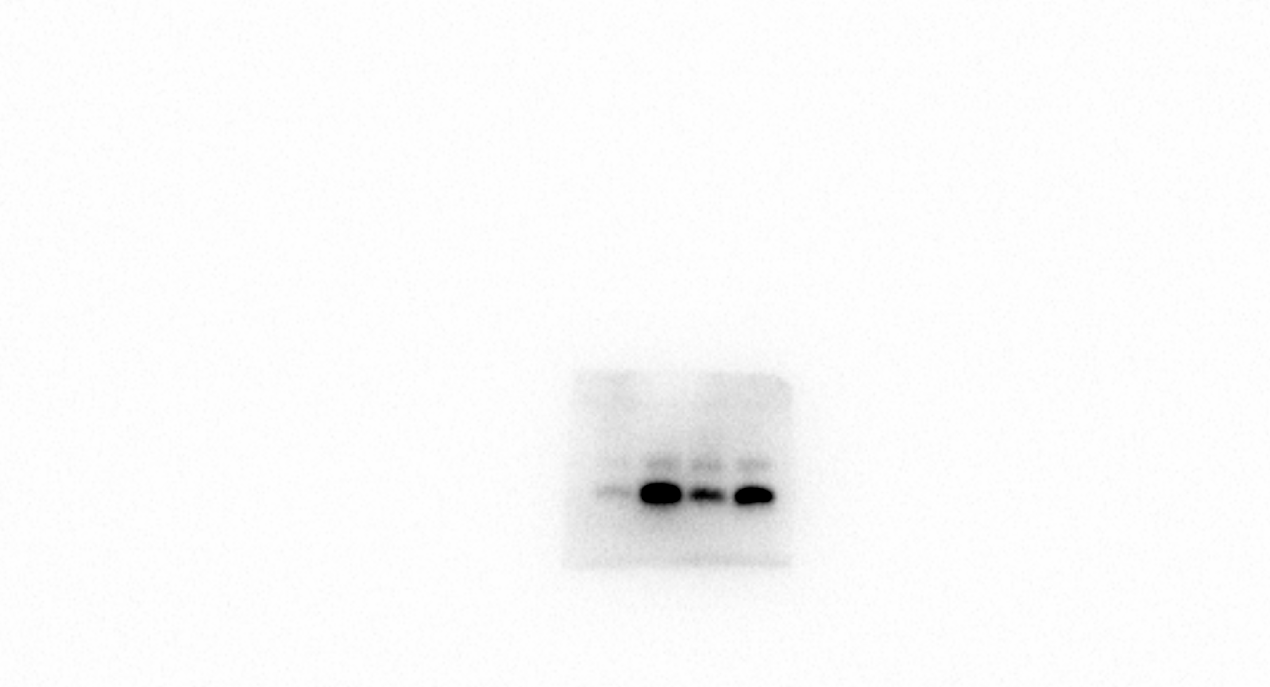

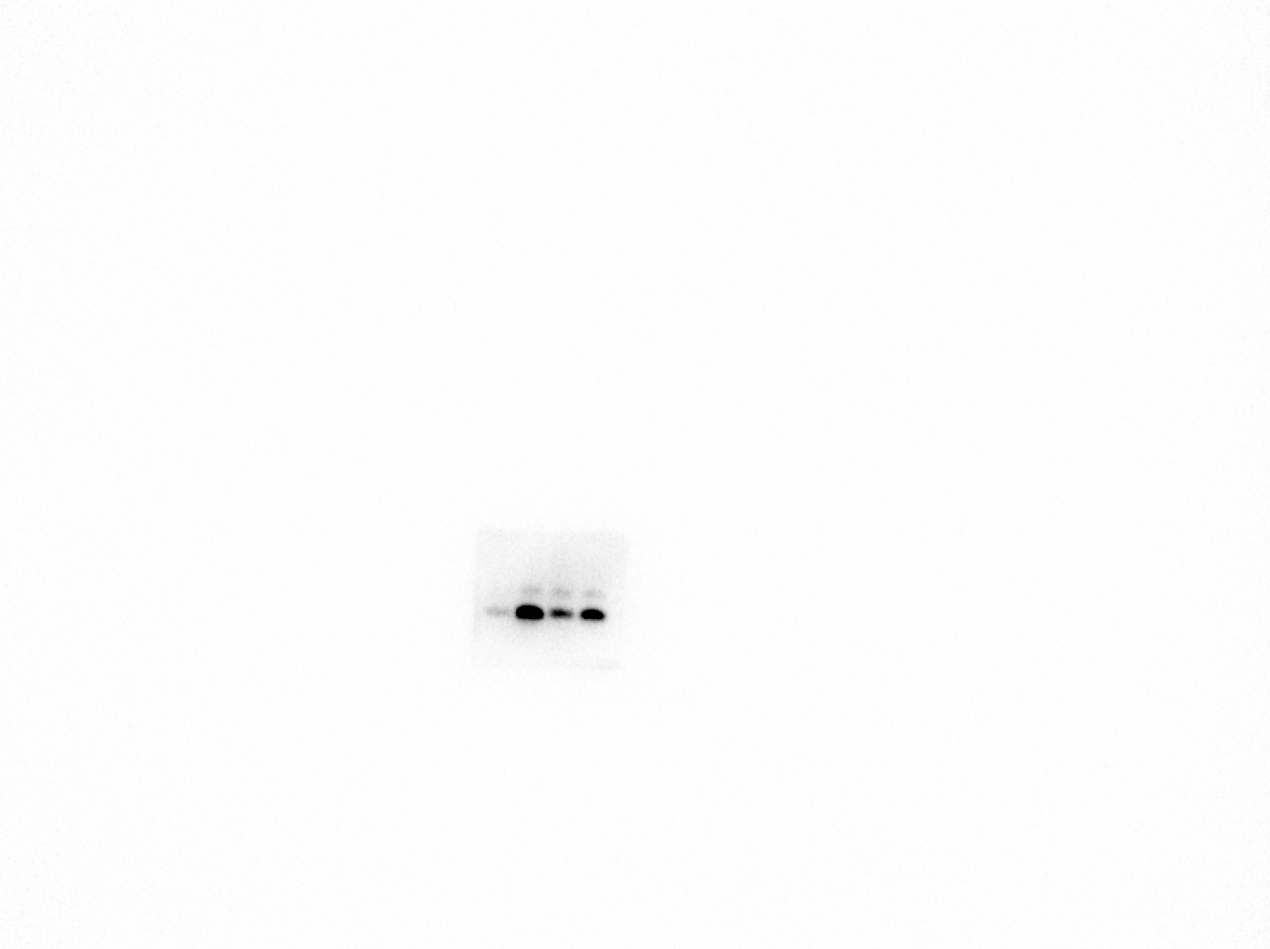


GAPDH
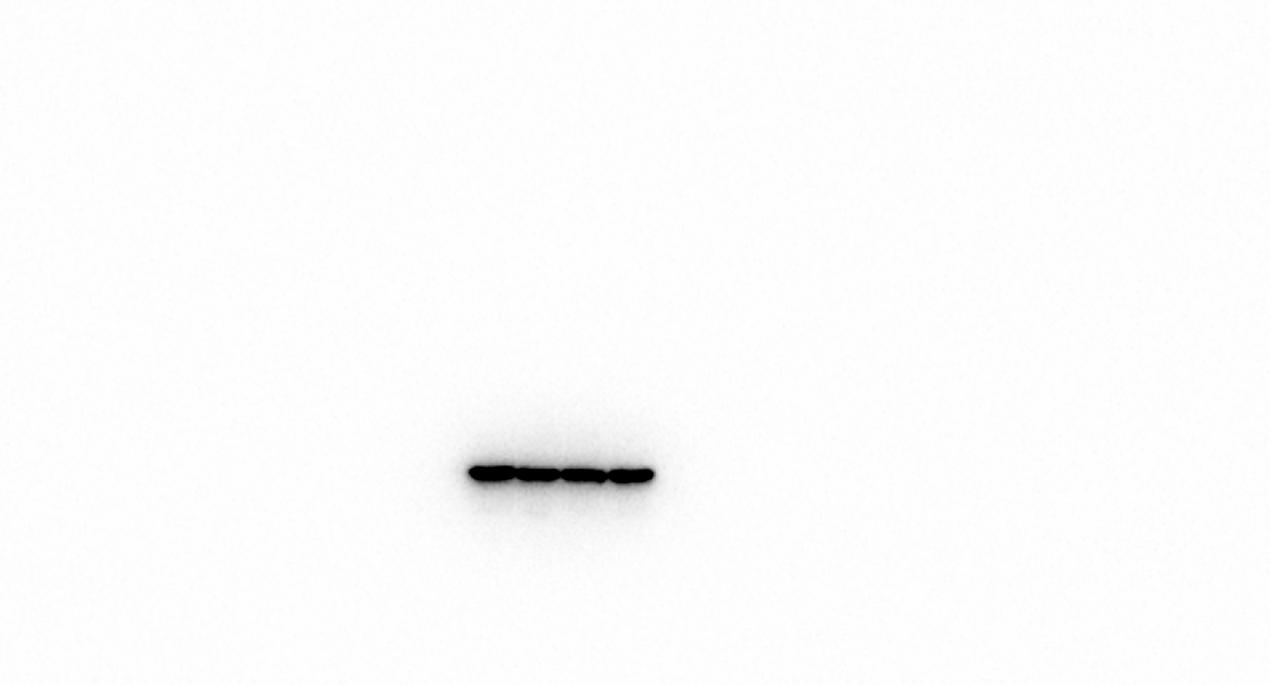

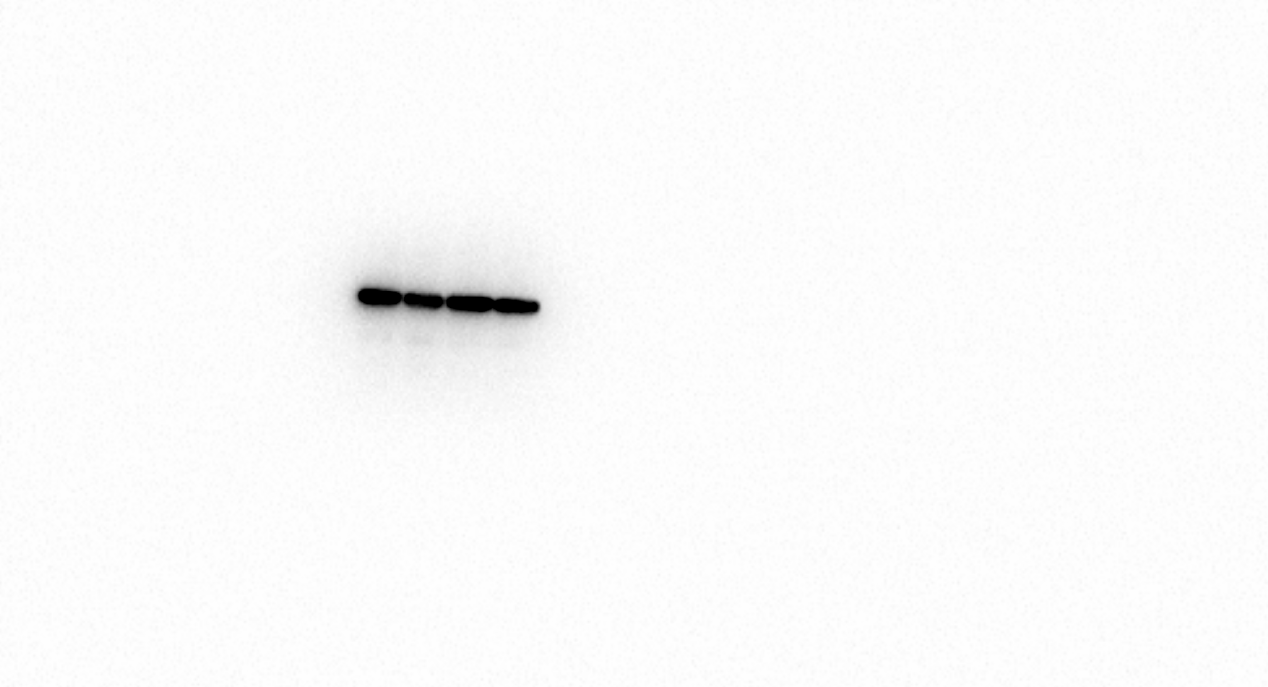

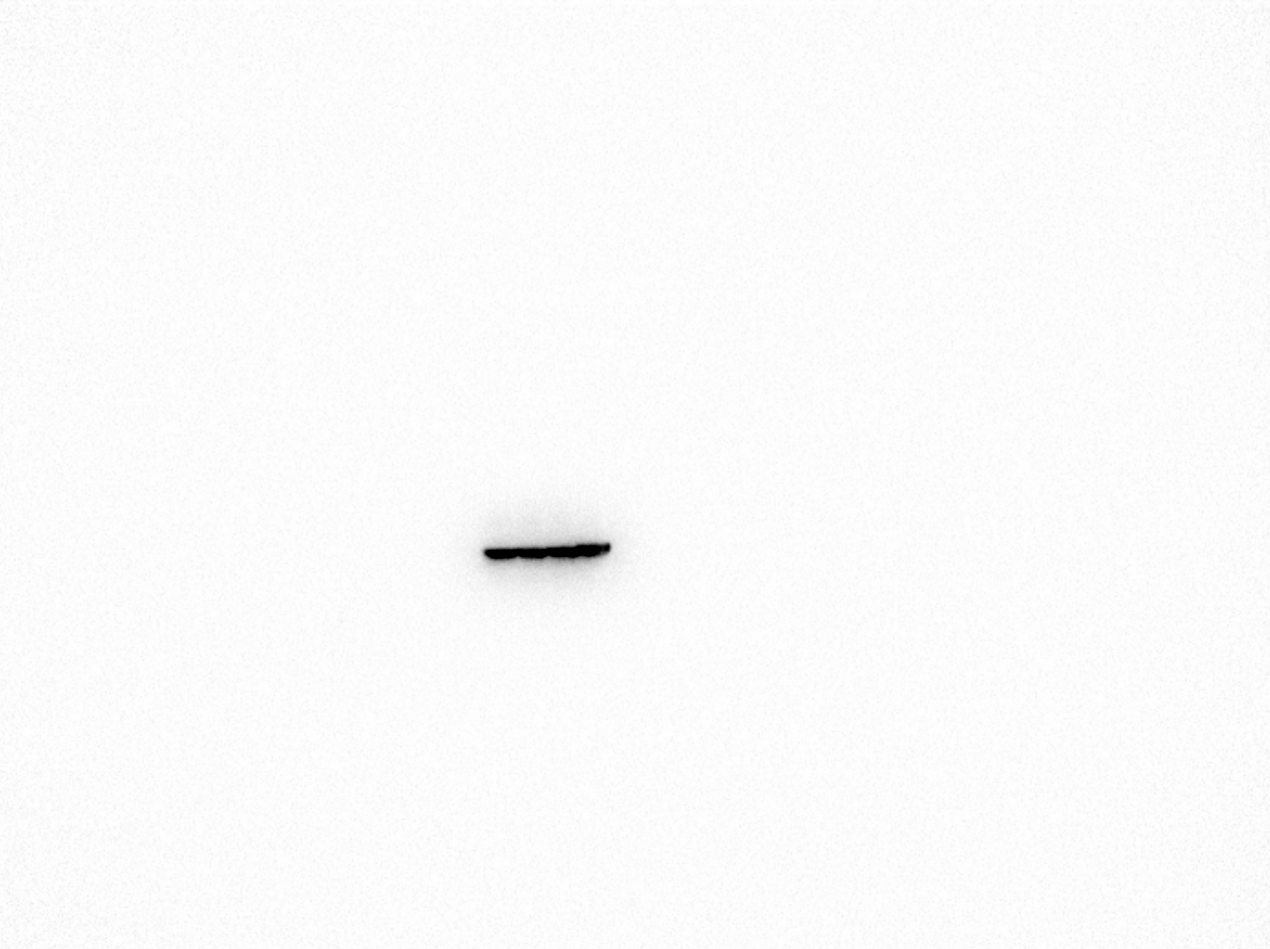


**Supplementary material7:**

**Figure 4G**

MKN45-CLDN18.2

PARP and Cleaved PARP
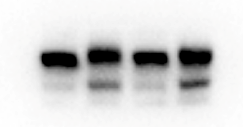

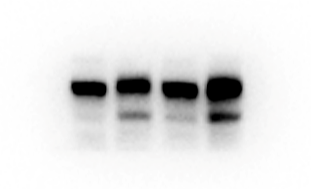

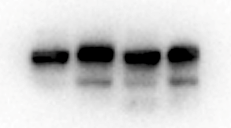


Caspase-9 and Cleaved caspase-9
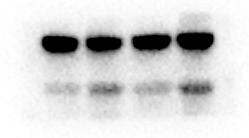

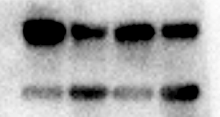

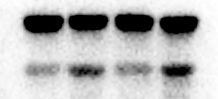


GAPDH
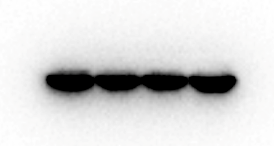

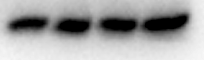

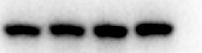


**Supplementary material8:**

**Figure 4H**

SNU-601

PARP and Cleaved PARP
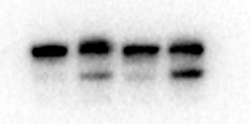

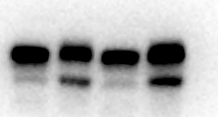

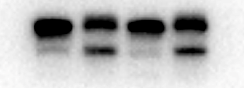


Caspase-9 and Cleaved caspase-9
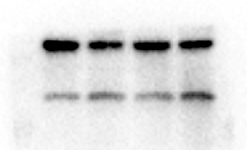

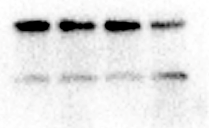

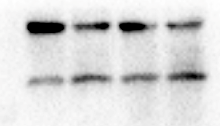


GAPDH
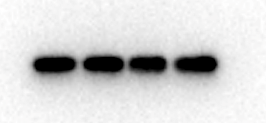

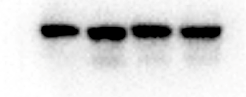

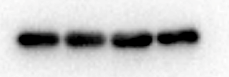


**Supplementary material9:**

**Figure 6A**

MKN45-CLDN18.2

p-mTOR-S2448
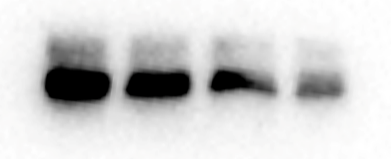

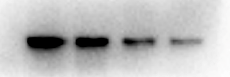

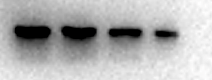


p-P70S6K-S371
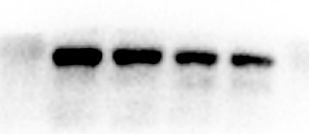

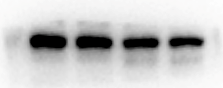

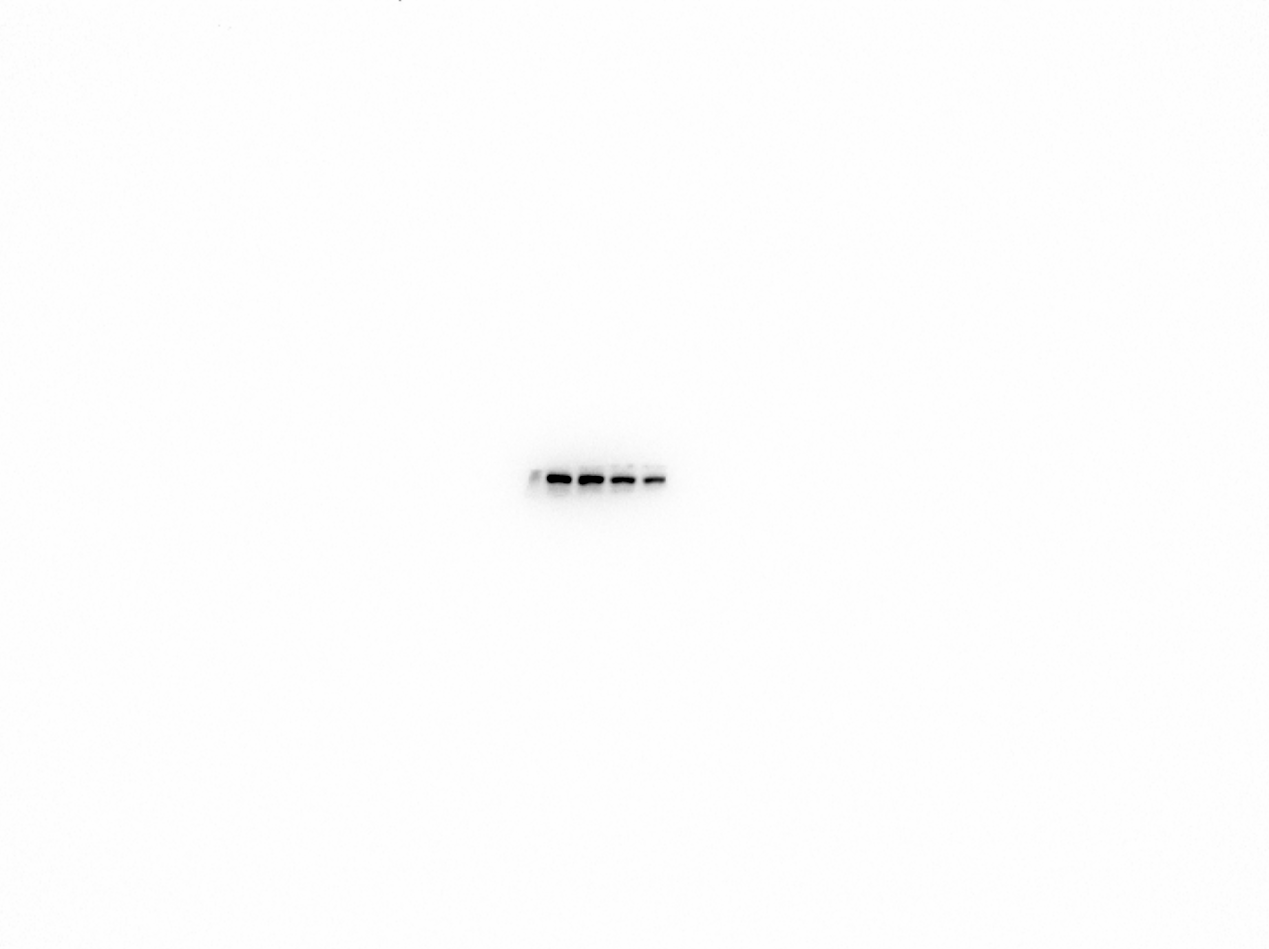


p-Akt-S473
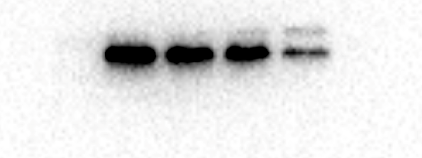

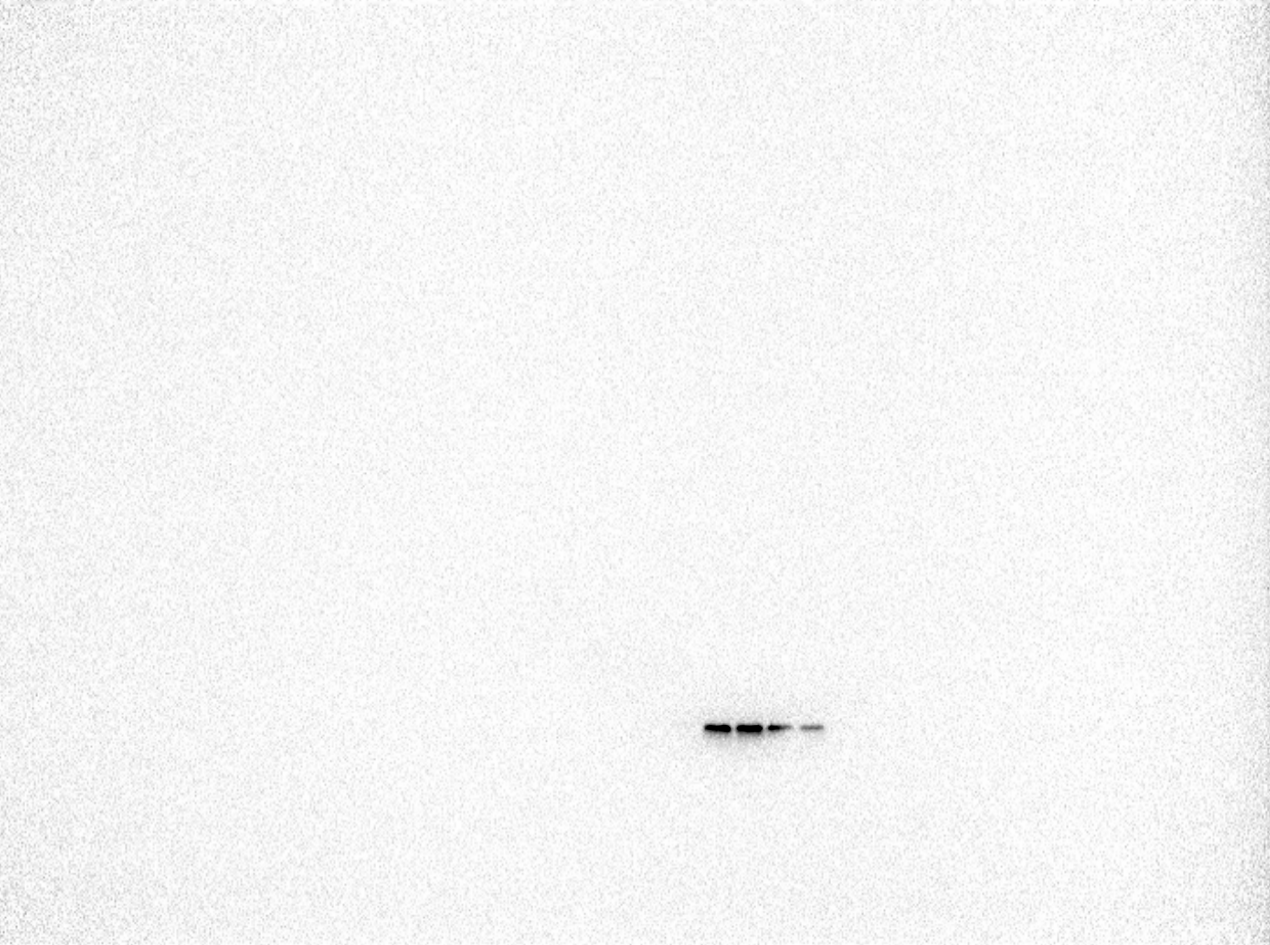

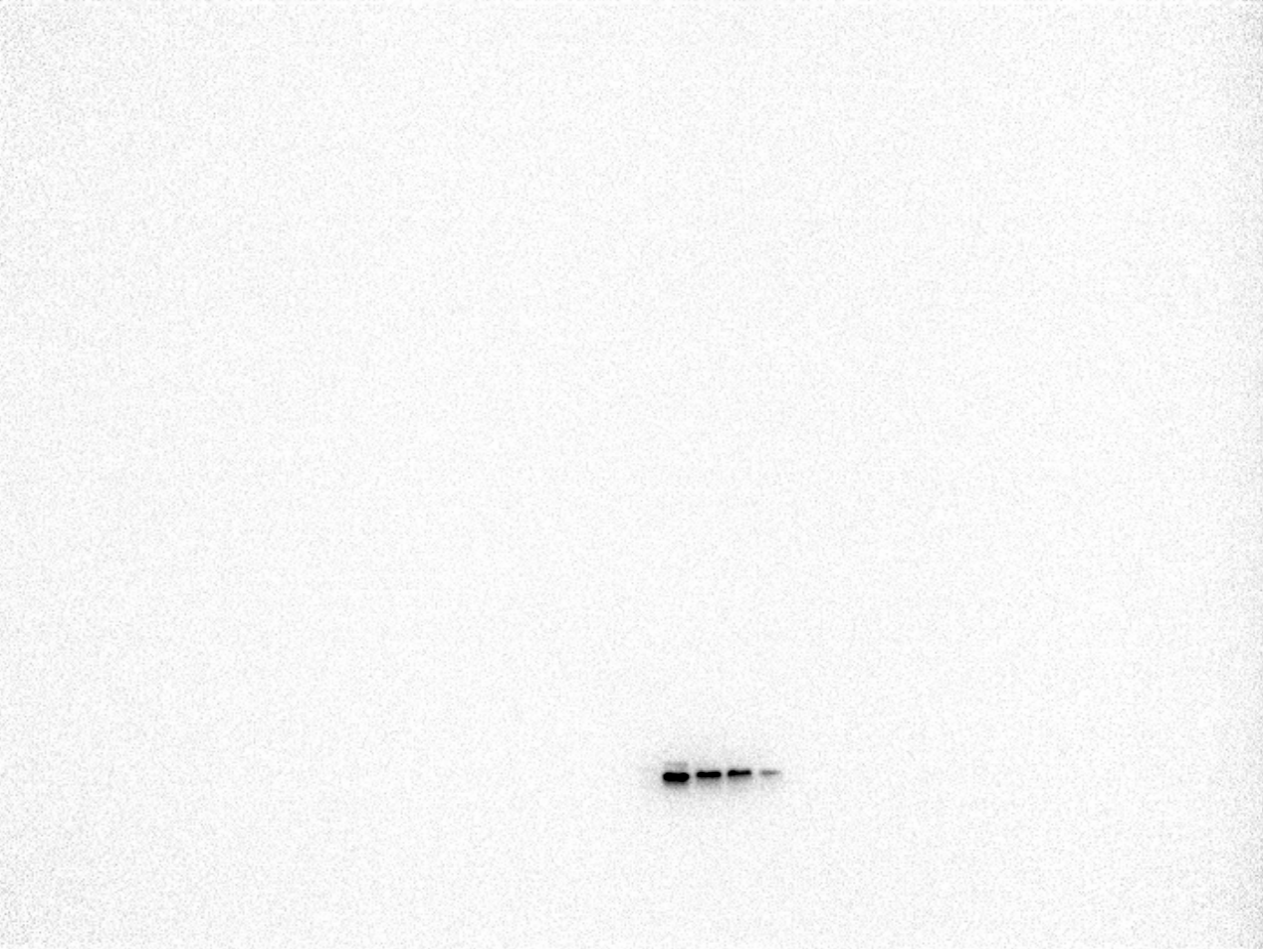


p-4E-BP1-T45
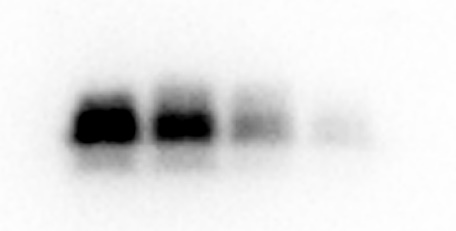

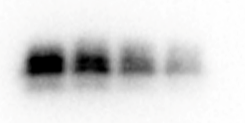

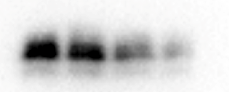


GAPDH
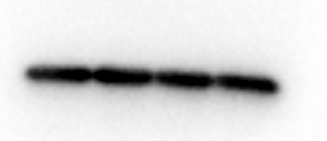

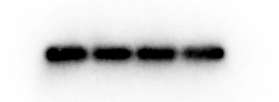

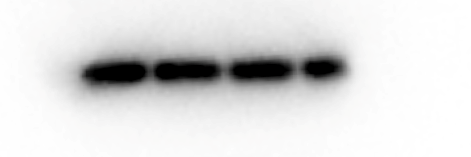


**Supplementary material10:**

**Figure 6C**

SNU-601

p-mTOR-S2448
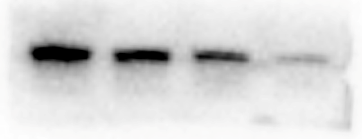

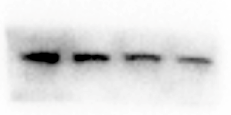

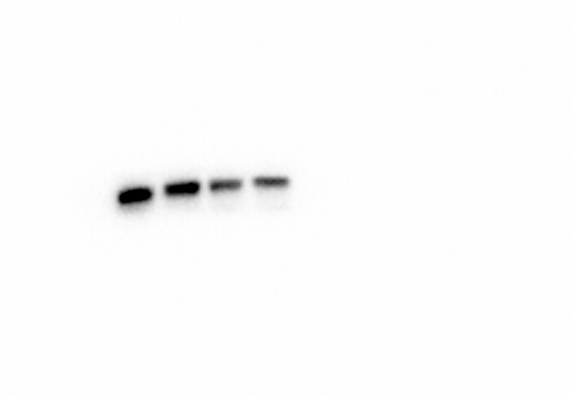


p-P70S6K-S371
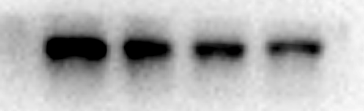

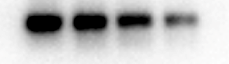

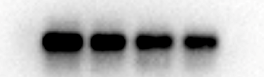


p-Akt-S473
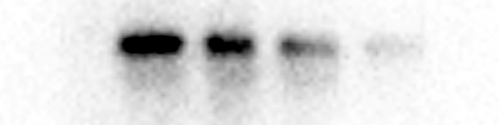

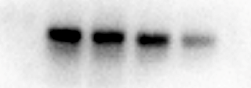

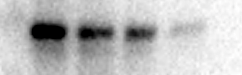


p-4E-BP1-T45
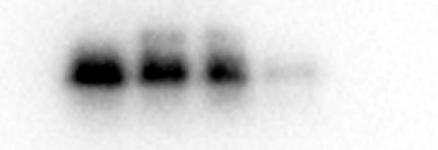

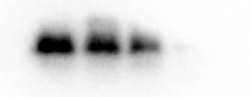

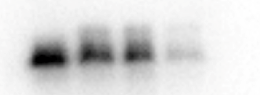


GAPDH
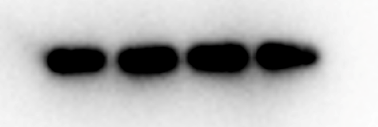

Supplement: Supplementary file 1 — Supplemental Material [file 41420_2024_2167_MOESM1_ESM.docx]
