# Supplementary material for: Enhancing antitumor efficacy of CLDN18.2-directed antibody-drug conjugates through autophagy inhibition in gastric cancer
Source: Cell Death Discov. 2024 Sep 3;10:393. doi: 10.1038/s41420-024-02167-0 (PMC11372199; doi:10.1038/s41420-024-02167-0)
Supplement: Supplementary file 2 — Supplementary Figure [file 41420_2024_2167_MOESM2_ESM.docx]

**Supplementary Figure**

**Enhancing Antitumor Efficacy of CLDN18.2-directed antibody-drug conjugates through Autophagy Inhibition in Gastric Cancer**

Wenjing Xue^1#^, Caili Xu^1#^, Kaiqi Zhang^2#^, Lu Cui^2^, Xiting Huang^1^, Yanyang Nan^1^, Dianwen Ju^1^*, Xusheng Chang^2^*, Xuyao Zhang^1^*

^1^Department of Biological Medicines & Shanghai Engineering Research Center of Immunotherapeutics, School of Pharmacy, Fudan University, Shanghai, 201203, China

^2^Department of Gastrointestinal Surgery, Changhai Hospital, Naval Medical University, Shanghai 200433, China

^#^These authors contributed equally to this work.

**Running title:** αCLDN18.2-MMAE and autophagy in gastric cancer

***Corresponding Author:**

Dianwen Ju, [dianwenju@fudan.edu.cn](mailto:dianwenju@fudan.edu.cn)

Xusheng Chang, [cxs20051014@163.com](mailto:cxs20051014@163.com)

Xuyao Zhang, [xuyaozhang@fudan.edu.cn](mailto:xuyaozhang@fudan.edu.cn)

**Supplementary Figure 1.**


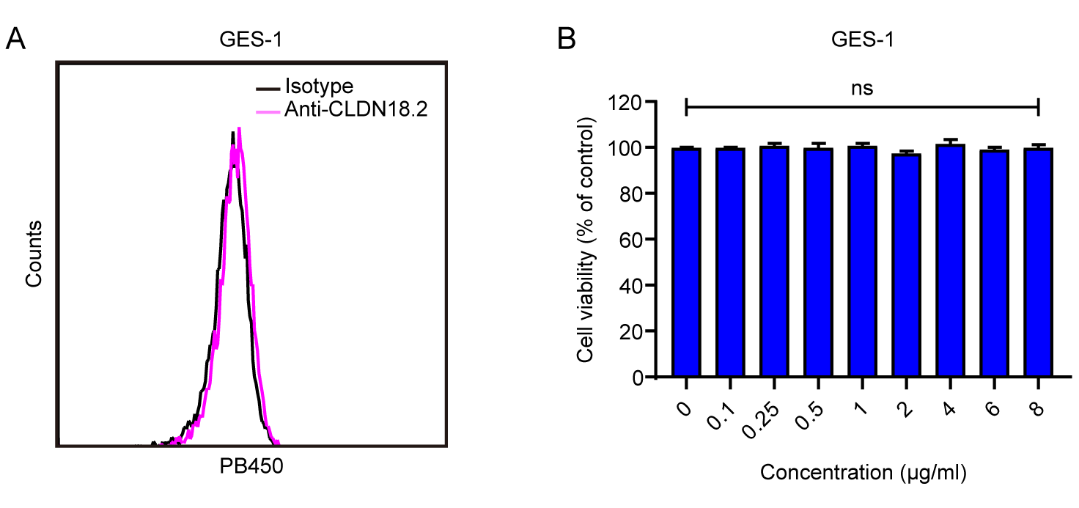


Figure S1. Cytotoxicity of αCLDN18.2-MMAE on GES-1 cells. A. Flow cytometry analysis of CLDN18.2 expression in GES-1 cells. B. Cell viability of GES-1 was evaluated using the CCK-8 assay following exposure to increasing concentrations of αCLDN18.2-MMAE for 48h. Data were shown as mean ± S.D. (n = 3) and analyzed by two-tailed unpaired t-test (ns not significant).

**Supplementary Figure 2.**


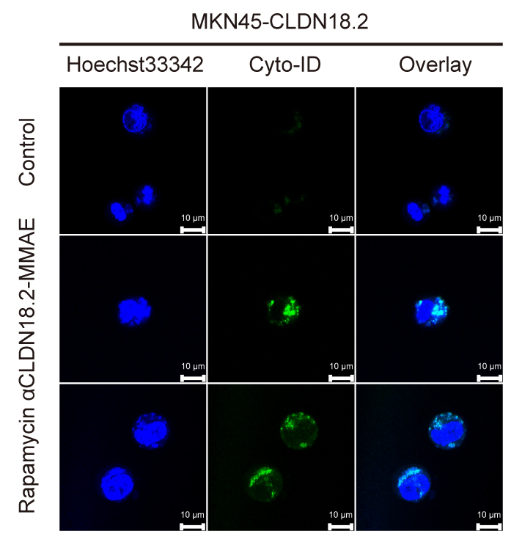


Figure S2. related to Figure 3. MKN45-CLDN18.2 cells were treated with αCLDN18.2-MMAE or Rapamycin for 48h. Cyto-ID and Hoechst 33342 were utilized to stain autophagosomes (green fluorescence) and cell nucleus (blue fluorescence), respectively.

**Supplementary Figure 3.**


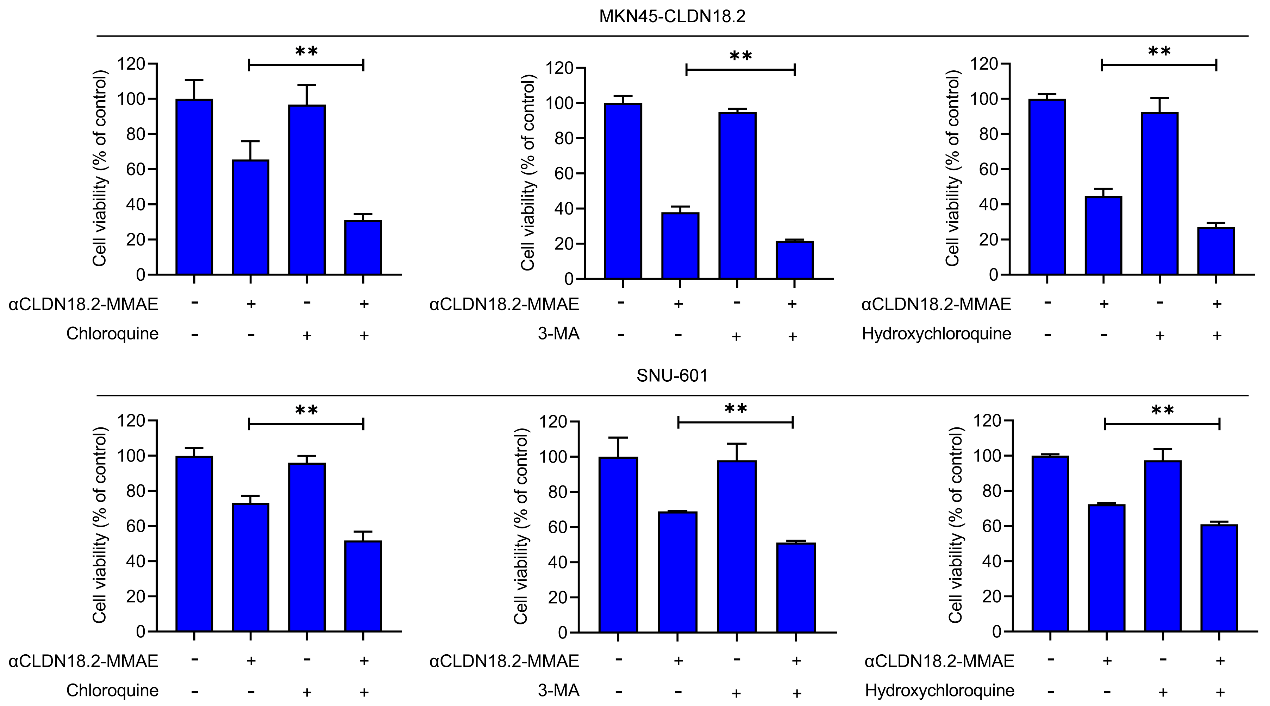


Figure S3. After treating MKN45-CLDN18.2 and SNU-601 cells with αCLDN18.2-MMAE (4 μg/mL) and either chloroquine (5 μM), 3-MA (0.125 mM), or hydroxychloroquine (0.5 μM for MKN45-CLDN18.2, 2.5 μM for SNU-601) for 48h, cell viability was evaluated using CCK-8 assay. Results were shown as mean ± S.D. (n = 3) and analyzed by two-tailed unpaired t-test (** *P* < 0.01).

**Supplementary Figure 4.**


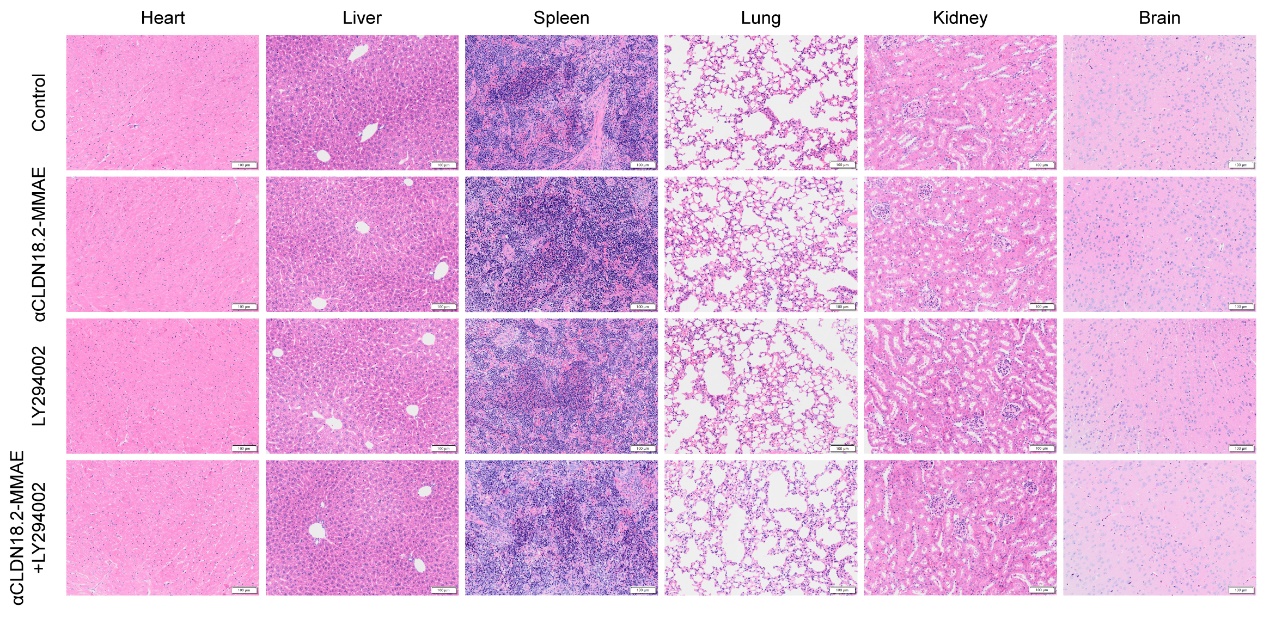


Figure S4. Representative H&E staining images of heart, liver, spleen, lung, kidney, and brain tissues from mice which were treated with PBS, αCLDN18.2-MMAE (2 mg/kg), LY294002 (50 mg/kg), or their combination twice a week for three weeks. Scale bar = 100 μm.
